# Influence of molecular structure on the antimicrobial function of phenylenevinylene conjugated oligoelectrolytes†
†Electronic supplementary information (ESI) available: supplementary MIC and confocal results, detailed synthesis pathways, organisms and culture conditions, and quantification of COE uptake by *E. coli* K-12 cells. See DOI: 10.1039/c6sc00630b


**DOI:** 10.1039/c6sc00630b

**Published:** 2016-06-01

**Authors:** Hengjing Yan, Zachary D. Rengert, Alexander W. Thomas, Carolin Rehermann, Jamie Hinks, Guillermo C. Bazan

**Affiliations:** a Department of Chemistry and Biochemistry , Center for Polymers and Organic Solids , University of California Santa Barbara , Santa Barbara , CA , USA . Email: bazan@chem.ucsb.edu; b Department of Chemistry , Ludwig-Maximilians-Universität München , Germany; c Singapore Centre for Environmental Life Sciences Engineering , Nanyang Technological University , Singapore . Email: jhinks@ntu.edu.sg; d Department of Materials , University of California Santa Barbara , Santa Barbara , CA , USA

## Abstract

Conjugated oligoelectrolytes (COEs) with phenylenevinylene (PV) repeat units are known to spontaneously intercalate into cell membranes. Twelve COEs, including seven structures reported here for the first time, were investigated for the relationship between their membrane disrupting properties and structural modifications, including the length of the PV backbone and the presence of either a tetraalkylammonium or a pyridinium ionic pendant group. Optical characteristics and interactions with cell membranes were determined using UV-Vis absorption and photoluminescence spectroscopies, and confocal microscopy. Toxicity tests on representative Gram-positive (*Enterococcus faecalis*) and Gram-negative (*Escherichia coli*) bacteria reveal generally greater toxicity to *E. faecalis* than to *E. coli* and indicate that shorter molecules have superior antimicrobial activity. Increased antimicrobial potency was observed in three-ring COEs appended with pyridinium ionic groups but not with COEs with four or five PV repeat units. Studies with mutants having cell envelope modifications indicate a possible charge based interaction with pyridinium-appended compounds. Fluorine substitutions on COE backbones result in structures that are less toxic to *E. coli*, while the addition of benzothiadiazole to COE backbones has no effect on increasing antimicrobial function. A weakly membrane-intercalating COE with only two PV repeat units allowed us to determine the synthetic limitations as a result of competition between solubility in aqueous media and association with cell membranes. We describe, for the first time, the most membrane disrupting structure achievable within two homologous series of COEs and that around a critical three-ring backbone length, structural modifications have the most effect on antimicrobial activity.

## Abstract 2

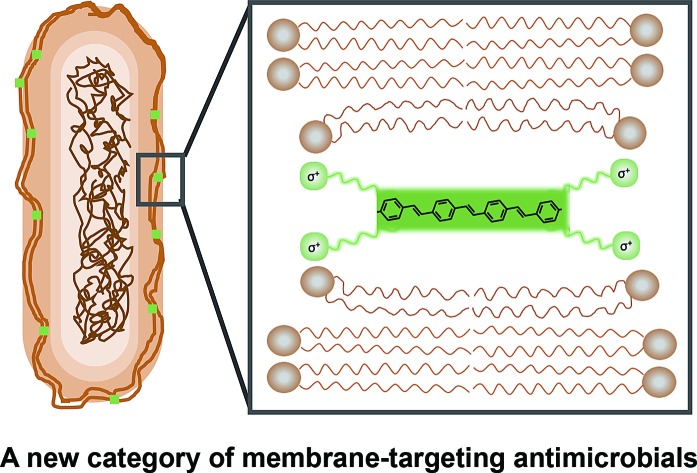
Structure/property relationships were obtained to understand the antimicrobial function of conjugated oligoelectrolytes toward Gram-negative and Gram-positive bacteria.

## Introduction

The ability of microbes to develop antimicrobial resistance underlies the emergence of drug resistant strains whose infections are increasingly difficult to treat, resulting in increased hospitalisation times with significant negative economic implications.^1^–^9^ New molecular systems to treat multidrug resistant strains that do not elicit microbial resistance are thus a research priority attracting significant scientific interest.^5^ Compounds whose antimicrobial activity arises from inserting into and disrupting biological membranes offer alternative strategies for the development of antimicrobials and have been the focus of recent studies.^6^–^19^

There are two major classes of antimicrobial membrane insertion (MIM) molecules: the naturally occurring antimicrobial peptides (AMPs) and synthetic mimics with cationic and/or amphiphilic properties.^10^–^13^ Compared to antibiotics, AMPs are less likely to induce resistance because of the non-specific mechanism of disruption.^14^,^15^ This disruption is thought to occur through membrane deformation and pore formation.^13^ Whilst the efficacy and microbial selectivity of AMPs are demonstrable, they have yet to find widespread utility clinically, partly due to high manufacturing costs.^13^ With similar proposed antimicrobial mechanisms, synthetic mimics, such as arylamide oligomers,^11^ polynorbornenes,^16^ and polymethacrylates,^17^ have the potential to overcome the manufacturing-costs and shelf life limitations of peptide-based counterparts.^18^,^19^ Moreover, some synthetic mimics, such as phenyleneethynylene-based conjugated polyelectrolytes,^13^,^20^ exhibit increased antimicrobial potency when activated by light offering enhanced treatment options.^21^

Conjugated oligoelectrolytes (COEs) with oligophenylenevinylene π-conjugated structural units have been reported to spontaneously intercalate into cell membranes. The spontaneous nature of this activity is thought to arise from the combination of a hydrophobic molecular backbone structure and terminal polar pendant-groups which resemble the charge distribution and hydrophobicity of phospholipids.^22^,^23^ The optical properties of an archetypical COE, *i.e*. 4,4′-bis(4′-(*N*,*N*-bis(6′′-(*N*′,*N*′,*N*′-trimethylammonium)hexyl)amino)-styryl)stilbene tetraiodide (**COE1-4**, also abbreviated in the literature as DSSN+), have been used to demonstrate that these molecules align within lipid bilayers with their long molecular axis normal to the membrane plane.^22^ With such high affinity for membranes, COEs have been applied in bioelectrochemical systems with different inoculum: wastewater,^24^ weak or model electroactive microbial species,^25^–^28^ and photosynthetic protein systems,^29^ and have been shown can significantly improve current generation or modify bioproduction yields.^30^ Electrochemical analysis suggest that COEs are not simply acting like electron shuttles, but rather they can couple metabolic intermediates with electrode surfaces, either directly or through increased mobilization across microbial membranes.^22^,^23^,^28^ More recently certain COEs have been shown to stabilize microbial membranes that have been subjected to butanol exposure.^31^

Comparison of structural modification by COEs with representative surfactant molecules, such as Tween 80 or 1,3-dimethyl-2-imidazolidinone (DMI), on mammalian membrane patches indicates that the intercalation of certain COEs does not necessarily destroy membranes.^32^ An assertion confirmed by the viability of *E. coli* retaining **COE1-4** in their membranes for over 20 generations.^33^ However, studies show that variations in the phenylenevinylene sequence length impact membrane integrity.^32^ Molecular dynamic simulations show that insertion of the three-ring cationic **COE1-3** results in membrane thinning as the lipid phosphate head groups are drawn toward the center of the bilayer.^34^ This membrane perturbation is less obvious upon intercalation of the four-ring cationic **COE1-4**, most likely due to a better match between its molecular length and the thickness of the lipid bilayer. Similarly, a three ringed COE with a fluorinated central aromatic ring (4F-DSBN+) exhibited a less-pronounced tendency for bilayer disruption.^23^,^34^,^35^ These findings indicate that molecular variations in COEs, especially in their aromatic content, and ionic charge density/distribution,^36^ can modulate their interactions with lipid membranes and the resulting structural perturbation. Accordingly, it seemed appropriate to probe relationships between COE molecular structures and their antimicrobial properties and to evaluate new COEs structures as antimicrobial MIMs.

**Chart 1:**
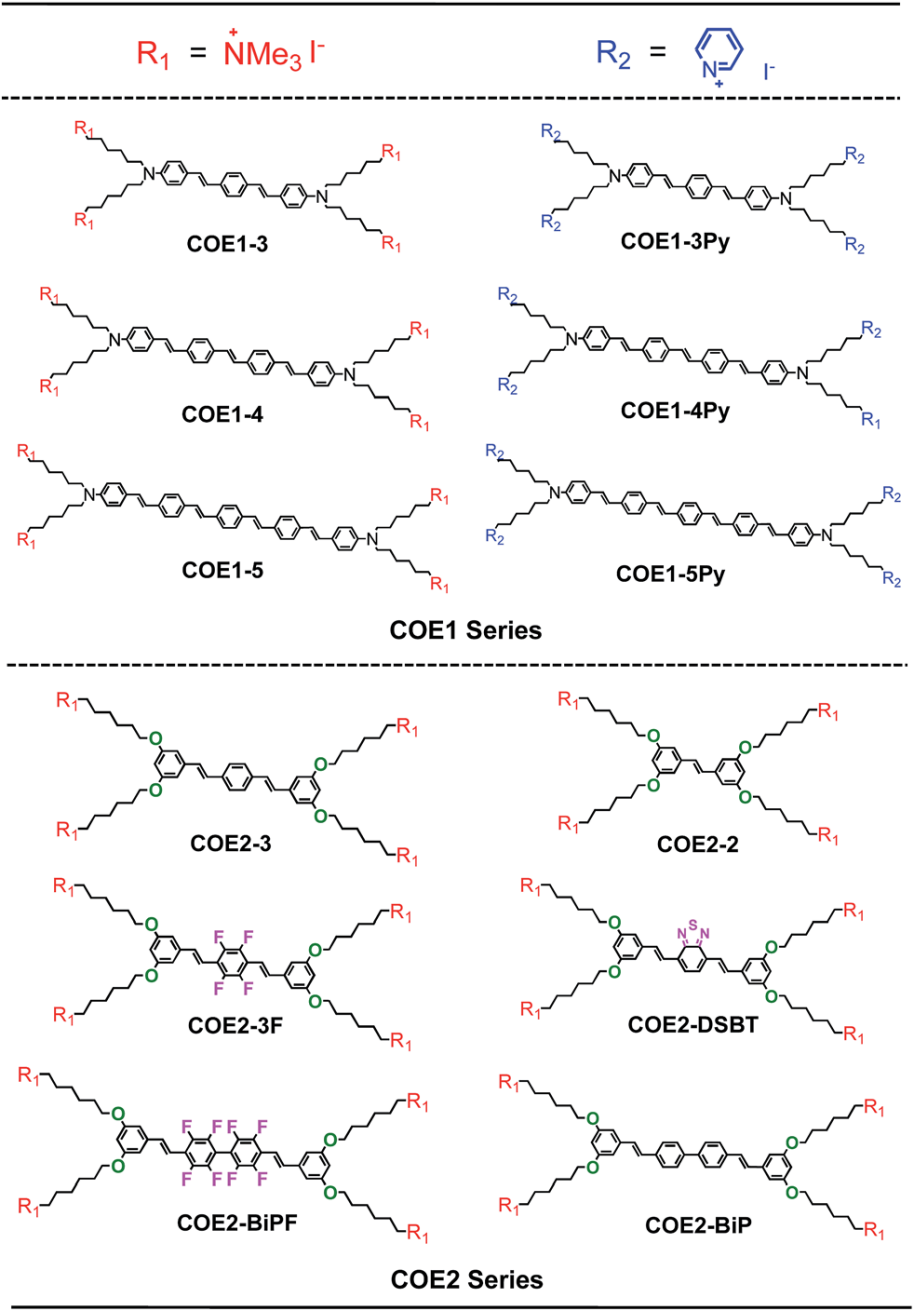
Molecule structures of 12 COEs tested in this study.

In this contribution, we report on a range of phenylenevinylene COE molecules (Chart 1) with structural variations in their backbone length, choice of ionic fragments and various central core modifications. Their antimicrobial properties are compared and discussed with respect to wild-type and mutant strains of a model Gram-negative bacterium (*Escherichia coli*) and a model Gram-positive bacterium (*Enterococcus faecalis*).^37^ Access to such a wide set of structurally related COEs reveals significant antimicrobial function and for the first time allows important structure–performance relationships to be definitively described.

The manuscript is organized as follows. We first introduce the chemical structures of the COEs under study, followed by the synthetic procedures for accessing seven new molecular designs. Next, we examine solvatochromic features to provide a baseline of optical signatures that are helpful to understand the extent of COE intercalation into cell membranes. Species information of microorganisms tested in this study along with and culture procedures are then provided. We follow by comparing the antimicrobial activity of COE structures, as determined by using minimal inhibitory concentration (MIC) tests, and a discussion of how COE structures impact toxicity.

## Results and discussion

### Molecular structures and synthesis

The list of compounds used in our studies is provided in Chart 1. They include variations in number of aromatic repeat units, *i.e.***COE1-3**, **COE1-4**, and **COE1-5**. One also finds pairs, such as **COE1-3** and **COE1-3Py**, which allow one to examine the impact of changing the cationic charged group from quaternary tetralkylammonium to the aromatic pyridinium fragment. There are also variations on the orientation of the solubilizing groups and the charge density within the conjugated framework, *i.e.***COE1-3***vs.***COE2-3**. Different aromatic fragments within the interior of the conjugated core have also been included, as in **COE2-DSBT** and **COE2-BiP**. Finally, the level of fluorination has also been included in the study by the inclusion of compounds **COE2-3F** and **COE2-BiPF**.

**Scheme 1:**
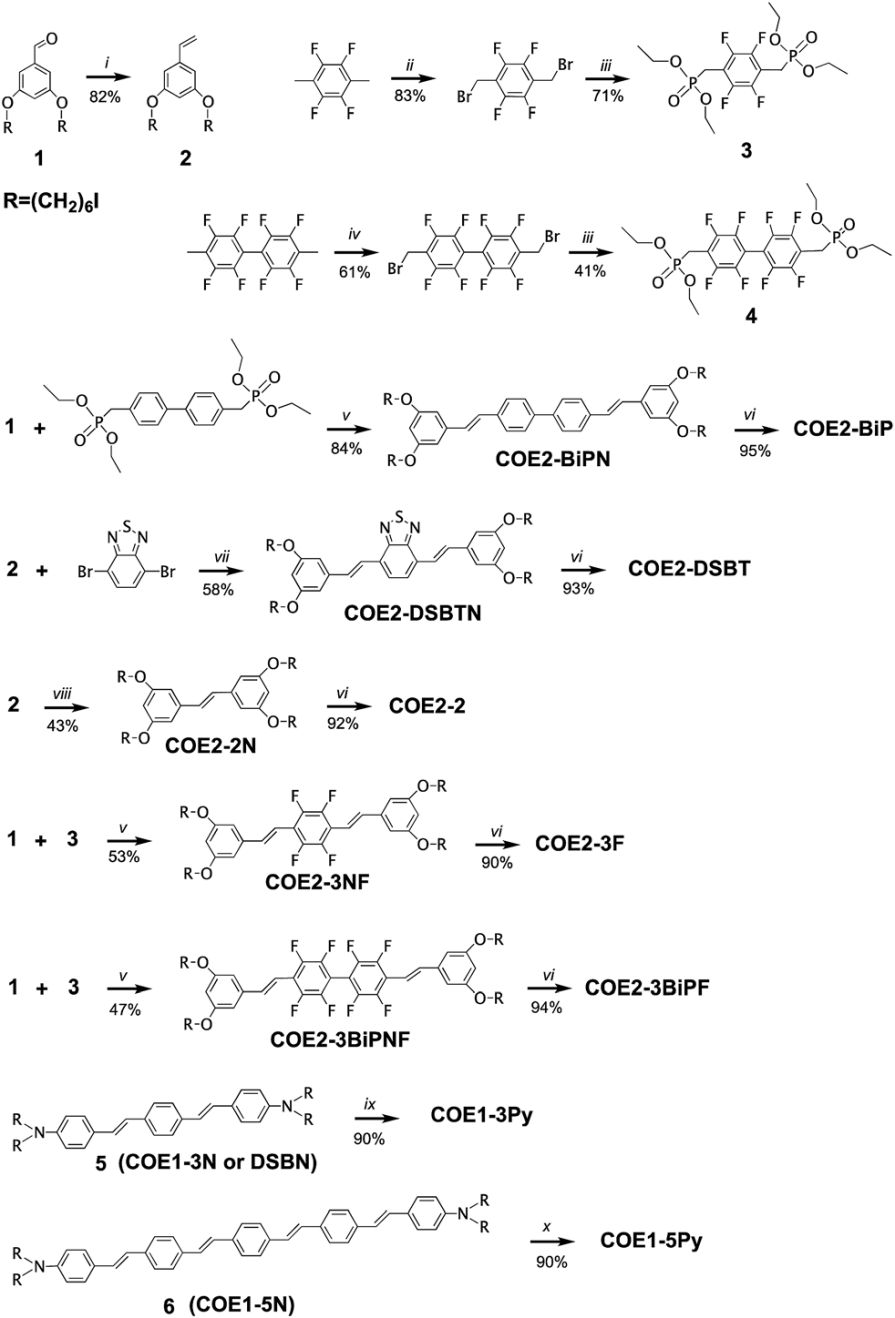
Synthetic schemes for the preparation of **COE1-3Py**, **COE1-5Py**, **COE2-2**, **COE2-3F**, **COE2-BiP**, **COE2-BiPF**, and **COE2-DSBT**^*a*^. ^*a*^Reagents and conditions: (i) CH_3_PPh_3_I, NaHMDS, 0 °C to RT, 2.5 h. (ii) NBS, benzoyl peroxide, DCM, *hν*, 18 h. (iii) P(OEt)_3_, toluene, reflux, argon atmosphere, 24 h. (iv) NBS, benzoyl peroxide, DCM, *hν*, 3 d. (v) KO^*t*^Bu, THF, RT, 24 h. (vi) (1) NMe_3_, THF, RT, 24 h; (2) NMe_3_, MeOH, RT, 24 h. (vii) (1) Pd(Oac)_2_, XPhos, Hünig's Base, toluene, 100 °C, 7 h. (2) NaI, acetone, reflux, overnight. (viii) Grubb's II, DCM, reflux, overnight. (ix) Pyridine, THF, MeOH, RT, 48 h. (x) Pyridine, THF, MeOH, 50 °C, 48 h. The preparations of 5 and 6 were described previously.^22^,^26^

The preparation methods and characterization of **COE1-3**, **COE1-4**, **COE1-5**, **COE2-3**, and **COE1-4Py** have been reported previously.^22^,^26^,^36^,^38^ Synthetic schemes for all the new COE molecules are provided in Scheme 1. Detailed procedures can be found in the ESI.† The key step in the synthesis of the new COEs involves formation of the internal π-conjugated system. This step can be accomplished *via* the use of Horner–Wadsworth–Emmons (HWE), Heck cross-coupling, or olefin metathesis reactions. Compound 1 was prepared as previously reported and its treatment under Wittig conditions afforded styrenyl derivative 2 in good yield.^26^ Compounds 3 and 4 were prepared from tetrafluoro-*p*-xylene and 2,2′,3,3′,5,5′,6,6′-octofluoro-4,4′-dimethyl-1,1′-biphenyl, respectively, after bromination of both benzylic positions, followed by an Arbuzov reaction utilizing triethylphosphite. Compounds 3 and 4 were then reacted with compound 1 under HWE conditions, to give the neutral fluorinated π-conjugated system in modest yield. Similarly, the **COE2-BiPN** derivative was prepared from the non-fluorinated biphenyl analogue of compound 4 under HWE conditions. The generation of **COE2-DSBTN** was accomplished *via* a Heck cross-coupling with compound 2 and 2,7-dibromobenzo-2,1,3-thiadiazole, while **COE2-2** was obtained from compound 2 by addition of Grubb's II catalyst to afford the two ring compound, both in yields of 58% and 43%, respectively. With all the neutral π-conjugated COE precursors in hand, ionization to give the final product was accomplished by quaternization of terminal alkylhalide groups by using either trimethylamine or pyridine. All of the products and intermediates were characterized using NMR spectroscopy and mass spectrometry, as described in the ESI.† The *trans* configuration of the olefins was confirmed on the basis of their coupling constants (*J* ≈ 16 Hz) in the ^1^H NMR spectra.

### Optical characterization

Solvatochromic features in the UV-Vis absorption and photoluminescence (PL) spectra were used to probe how the molecular structure responds towards the polarity of the medium. These studies provide a basis for determining the extent to which each COE inserts into the membrane. Of particular use is the availability of the neutral and charged versions of the same optically active fragment to record spectra in toluene and water, respectively. For example, the features of **COE1-3N** in toluene were compared against those of **COE1-3Py** in water. The results of these studies are summarized in Table 1. Absorbance maxima (*λ*_abs_) range from 306 nm to 448 nm for all the COEs. Small shifts of *λ*_abs_ were found in most COE molecules tested here when one changes the solvent from toluene to water, however, this shift in absorption is not apparent in **COE1-3Py** or **COE1-5**. In COE1 Series, replacing tetraalkylammonium ending groups with pyridinium results in smaller molar extinction coefficients (*ε*_max_) at *λ*_max_. Since smaller *ε*_max_ values have been reported for the COE1 series when measured in water instead of toluene, we assume a similar solvatochromic effect with the pyridinium functionalization. Differing from the other COEs, **COE2-DSBT** and its neutral version **COE2-DSBTN** exhibit *λ*_abs_ at more red-shifted wavelengths (448 nm and 443 nm), which is consistent with the electron deficiency of the benzothiadiazole and its impact on the charge transfer characteristics of the excited state.

**Table 1:** Summary of UV-Vis and PL spectra of COE molecules in toluene and water. Molar extinction coefficients (*ε*_max_, L mol^–1^ cm^–1^) were measured at *λ*_max_

	Toluene		Water	
	*λ*_abs_ (*ε*_max_ × 10^4^)	*λ*_em_	*λ*_abs_ (*ε*_max_ × 10^4^)	*λ*_em_
**COE1-3N**	410 (7.1)	453^*b*^, 485		
**COE1-3** ^*a*^			404 (6.0)	566
**COE1-3Py**			410 (3.8)	560
**COE1-4N** ^*a*^	425 (10.7)	476		
**COE1-4** ^*a*^			412 (6.6)	594
**COE1-4Py**			415 (3.8)	N/A
**COE1-5N**	430 (10)	482^*b*^, 510		
**COE1-5**			429 (9.3)	593
**COE1-5Py**			420 (2.6)	543^*c*^
**COE2-2N**	310 (9.8)	355^*b*^, 373		
**COE2-2**			306 (3.4)	425
**COE2-3NF**	324 (3.0)	395^*b*^, 422		
**COE2-F**			335 (2.8)	438
**COE2-BiPN**	355 (7.4)	403^*b*^, 426		
**COE2-BiP**			348 (6.6)	426
**COE2-BiPNF**	335 (6.1)	393		
**COE2-BiPF**			328 (3.5)	452
**COE2-DSBTN**	448 (1.8)	550		
**COE2-DSBT**			443 (2.5)	N/A

^*a*^From Garner *et al.*^22^

^*b*^Within the two maxima emission wavelengths, the more blue-shifted *λ*_em_ is always more intense for related compounds in this table. More specifically, the intensity ratio of two *λ*_em_ is between 1.2 and 1.5.

^*c*^This measured PL result has a low signal to noise ratio due to a low solubility of **COE1-5Py** in water.

Red shifts of 33–75 nm can be observed in the PL maxima (*λ*_em_) of **COE1-3Py**, **COE1-5Py**, **COE2-2**, and **COE2-BiPF** in water, compared to their corresponding neutral precursors **COE1-3N**, **COE1-5N**, **COE2-2N**, and **COE2-BiPNF** in toluene (Table 1), consistent with previous findings in **COE1-3**.^22^ However, the PL signals from **COE1-4Py** were below detection using a standard fluorometer. For **COE2-DSBTN**, *λ*_em_ is also red-shifted compared to the other COEs at 550 nm. The PL signal of the charged species **COE2-DSBT** is found to be quenched in water, showing that this molecule has a strong sensitivity to the polarity of the surrounding medium. Such hypsochromic shifts, together with increased quantum efficiencies of COEs in less polar solvent environment,^22^ allow us to estimate the extent of COE intercalation into lipid membranes from aqueous solution using UV-Vis absorption and PL spectroscopies, while confocal fluorescent microscopy offers qualitative confirmation of these observations.

### Organisms and culture conditions

*Escherichia coli* K-12 was chosen as a representative Gram-negative organism and to align our work with previous studies.^33^*E. coli* WBB06, a mutant having a defective outer membrane and its parental type, W3110,^39^–^41^ were chosen, as they have been previously shown to be useful for studying if antibiotics are excluded by the outer membrane. *E. coli* were grown overnight from single colonies in LB medium unless otherwise noted. *Enterococcus faecalis* was chosen as a representative Gram-positive pathogen that is clinically significant and implicated in drug resistant nosocomial infections and, similarly, to align our data with previous studies.^42^,^43^ The organisms used here were *E. faecalis* OG1X and a mutant (*ΔdltA-D*) that is unable to express d-alanylated teichoic acids in its cell wall. The resulting mutant has an overall more negative net charge on the bacterial cell surface than wild-type *E. faecalis* OG1X. This allows us to investigate electrostatic interactions between COEs and the microbial envelope. *E. faecalis* cultures were grown in brain heart infusion medium. A summary of the organisms used in this study is provided in Table S1.†

### COE uptake by cells

Absorption spectroscopy was used to estimate the extent to which COEs accumulated in *E. coli* K-12 membranes. Specifically, UV-Vis absorption was used to quantify the concentrations of 5 μM COE remaining in solution after incubation with *E. coli* (ESI†). The results are summarized in Table 2. Briefly, cultures of *E. coli* K-12 (adjusted to OD = 1.0) were collected by centrifuge, and washed twice with phosphate buffered saline (PBS) solution (pH = 7.2). The cells were resuspended in PBS and treated with 5 μM COE for 1.5 hours at room temperature (S3 in ESI†). Resulting incubations were centrifuged once more and the supernatant was collected for absorbance scans using a plate reader to quantify the remaining COE. COE concentration in each sample was calculated according to the linear relationship between absorbance values and COE concentrations obtained from standards of each COE at 0, 2.5, 5, and 10 μM in PBS solution (Fig. S1 and S2†). Treated cells were collected and resuspended in 1 × PBS solution (adjusted to OD = 1.0) for absorbance scans and confocal microscopy imaging. To exclude the background interference introduced by the addition of *E. coli*, absorbance signals from supernatant and biological cell samples prepared identically but without adding COEs were collected separately and used as blanks (Fig. S3†).

**Table 2:** Summary of relative COE uptake by *E. coli* K-12 cells as determined by UV-Vis spectroscopy. (± = 1 × standard deviation, *n* = 2)

Molecule	Abs by cells (%)	Molecule	Abs by cells (%)
**COE1-3**	75 ± 5	**COE2-2**	B.D.^*a*^
**COE1-3Py**	63 ± 6	**COE2-3**	27 ± 8
**COE1-4**	75 ± 3	**COE2-3F**	65 ± 6
**COE1-4Py**	72 ± 5	**COE2-Bip**	85 ± 5
**COE1-5**	80 ± 7	**COE2-BipF**	70 ± 3
**COE1-5Py**	78 ± 7	**COE2-DSBT**	66 ± 4

^*a*^B.D.: below detection limit.

**Fig. 1:**
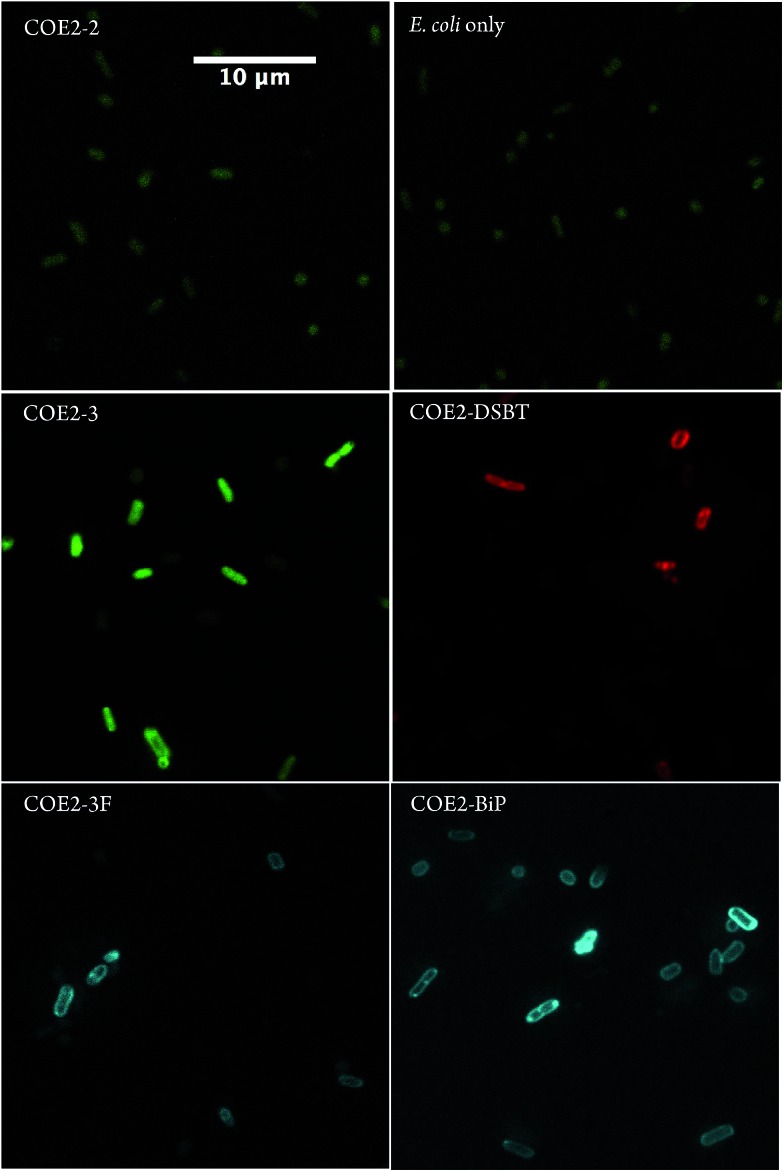
Confocal microscopy images of *E. coli* K-12 cells treated with 5 μM of **COE2-2**, **COE2-3**, **COE2-3F**, **COE2-BiP**, and **COE2-DSBT** for 1.5 hours in PBS buffer at room temperature. Auto-fluorescent signals from *E. coli* cells were collected with the same detection settings for **COE2-2** panel. Scale bar is the same for all panels. Laser excitation at 405 nm).

From Table 2, the UV-Vis absorbance measurements reveal significant uptake (>60%) of all structures within the COE1 series by *E. coli* K-12. Uptakes for the COE2 series were estimated between 27% and 85%, with the notable exception of **COE2-2**, which remained mostly in solution. In agreement with the UV-Vis data, confocal fluorescence microscopy showed membrane accumulation of all the COEs in Table 2, except for **COE2-2**, within cell membranes (Fig. 1, S4, and S5†). Of particular interest is that **COE2-2** did not appear to accumulate into *E. coli* to any significant degree (Table 2). Confocal fluorescent signals from **COE2-2**-treated cells were at a similar level with autofluorescence from non-treated cells (Fig. 1 and S5†). This unusually low uptake likely results from its higher solubility in water, because of the higher ratio of charged end groups relative to the hydrophobic backbone, compared to the other 11 COE structures. From the data in Table 2, one can detect a general trend of increased membrane accumulation with increased backbone length, from **COE2-2**, **COE2-3**, to **COE2-BiP**. As a result, slight changes in the amphiphilic properties of COE structures are anticipated to modulate affinity to biological cell membranes in aqueous solution, which might in turn affect their antimicrobial activity.

### Antimicrobial tests

**Fig. 2:**
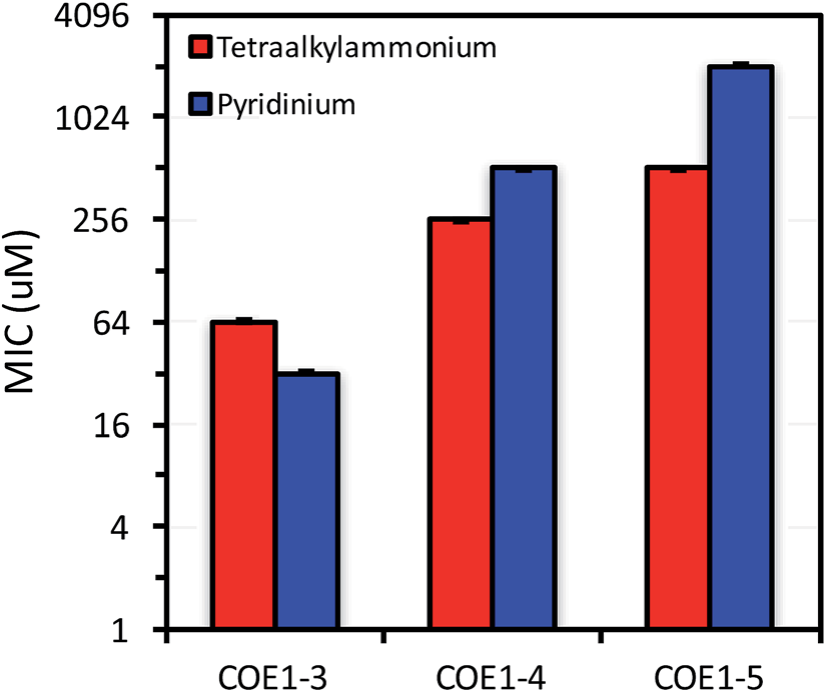
MIC of **COE1-3**, **COE1-4**, and **COE1-5** on *E. coli* K-12 with tetraalkylammonium or pyridinium end groups. The *Y*-axis is in log_2_ scale for a better display of the results obtained from 2-fold dilution method.

**Fig. 3:**
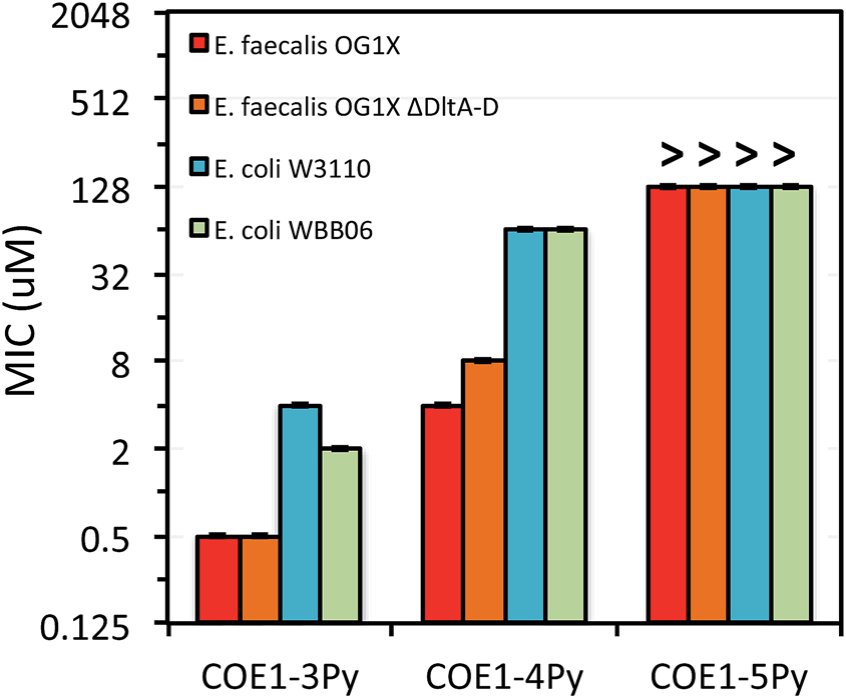
MIC of **COE1-3Py**, **COE1-4Py**, and **COE1-5Py** on *E. coli* W3110, *E. coli* WBB06, *E. faecalis* OG1X, *E. faecalis* OG1X *ΔdltA-D*. (“>” refers to a MIC result larger than the highest concentration we tested). The *Y*-axis is in log_2_ scale for a better display of the results obtained from the 2-fold dilution method.

**Fig. 4:**
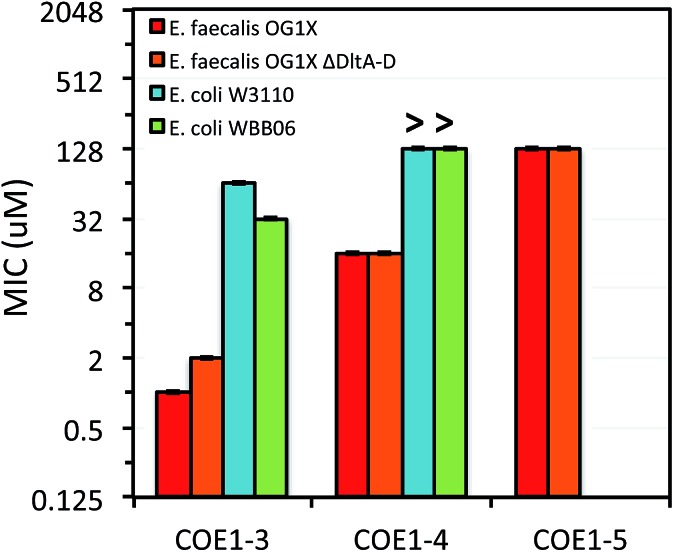
MIC of **COE1-3**, **COE1-4**, and **COE1-5** on *E. coli* W3110, *E. coli* WBB06, *E. faecalis* OG1X, *E. faecalis* OG1X *ΔdltA-D*. (“>” refers to a MIC result larger than the highest concentration we tested). The *Y*-axis is in log_2_ scale for a better display of the results obtained from the 2-fold dilution method. MIC test of **COE1-5** on *E. coli* W3110, *E. coli* WBB06 was not performed because MICs of **COE1-4** on the two bacterial strains are already over 128 μM.

Minimum inhibition concentration (MIC) tests using a broth microdilution method were used to determine the lowest COE concentration that inhibits bacterial growth.^44^ Briefly, COEs were diluted *via* a 2-fold dilution series in LB medium to final concentrations ranging from 4096 μM to 0.25 μM in a 96-well plate. Each well was inoculated with 5 × 10^5^ CFU mL^–1^ of the respective organism. Cell densities in culture were monitored by measuring OD_600 nm_. Inoculum densities were standardized from a predetermined relationship between optical density and cell counts and verified by direct plate counting techniques for each test. MIC tests were carried out in triplicate. MIC values were determined as the lowest COE concentration that prevents visible microbial growth in the medium over a period of 12 h at 37 °C.^45^ The results of these studies are summarized in Fig. 2–4, and in Table 3. A discussion of how different structural variables influence MIC follows.

**Table 3:** MIC of **COE2-2**, **COE2-3**, **COE2-BiP**, and **COE2-BTDA** (no fluorine substitution) and **COE2-3F** and **COE2-BiPF** (fluorine substitution on backbone) on *E. coli* K-12. Standard deviations between duplicates are negligible due to the 2-fold dilution method

Molecule	MIC (μM)
**COE2-2**	128
**COE2-3**	8
**COE2-3F**	2048
**COE2-BiPF**	2048
**COE2-BiP**	512
**COE2-DSBT**	512

#### Backbone length of the COE1 series

As shown in Fig. 2, MIC values on *E. coli* K-12 increase from **COE1-3** to **COE1-5**. This trend is observed when these molecules contain either tetraalkylammonium or pyridinium end groups (Fig. 2). Shortening the conjugated sequence length in this series of compounds therefore leads to higher antimicrobial activity. This trend remains true for both Gram-positive and Gram-negative organisms including *E. coli* W3110, *E. coli* WBB06, *E. faecalis* OG1X, and *E. faecalis* OG1X *ΔdltA-D* (Fig. 3). Biological uptake of the COE1 series is not significantly affected by the conjugated backbone length (Table 2). This supports that shorter COEs are more toxic mainly due to their stronger disturbing effect on membrane stability rather than their greater membrane accumulation in quantity.^34^

Previous molecular dynamic simulations indicate that membrane deformation may occur upon COE insertion as the lipid phosphate head groups are drawn toward the center of the bilayer by electrostatic force. Such distortions are more pronounced when the molecular length is shorter than the thickness of lipid bilayer.^34^ From **COE1-5** to **COE1-3**, one would expect the membrane thinning effect on lipid bilayers to decrease progressively. Our results show that shortening the COE1 series backbone length from five aromatic rings to three aromatic rings results in a more toxic antimicrobial structure (Fig. 2–4), and the greater toxicity of shorter COE is not due to more uptake by cells (Table 2). These trends are consistent with the previously proposed mechanisms derived from molecular dynamic simulations.^34^

#### Ionic pedant groups in the COE1 series

Replacing tetraalkylammonium cationic end group with pyridinium did not significantly affect molecular uptake by cells. Overall only a 2–12% difference was observed in uptake by cells of COEs with the two different cationic groups (Table 2). In MIC tests, replacing tetraalkylammonium with pyridinium leads to lower toxicity of **COE1-4Py** and **COE1-5Py** toward *E. coli* K-12. For example, MIC changes from 256 μM (**COE1-4**) to 512 μM (**COE1-4Py**), and 512 μM (**COE1-5**) to 2048 μM (**COE1-5Py**). However, for **COE1-3**, this replacement results in the most toxic structure within the COE1 series (MIC = 32 μM for **COE1-3Py**, Fig. 2). In fact, the increased toxicity from **COE1-3** to **COE1-3Py** is observed for the other bacterial strains tested in this study, suggesting that **COE1-3Py** is the most toxic structure achievable currently within the COE1 series and that the cationic terminal group should be a relevant structural unit for modification in further studies. **COE1-3Py** has a particularly high activity against *E. faecalis* with a MIC of 0.5 μM (Fig. 3 and 4). Slight increased toxicity from **COE1-4** to **COE1-4Py** is also found for *E. coli.* WBB06, *E. coli.* W3110, *E. faecalis* OG1X, and *E. faecalis* OG1X *ΔdltA-D*. No increased toxicity is found from **COE1-5** to **COE1-5Py**, in any bacterial strain tested in this study (Fig. 2–4). These observations indicate that molecular modifications have the most impact when the backbone length of the COE is inherently antimicrobial in nature *e.g.*, in this instance, **COE1-3Py**, and **COE1-4Py**. This insight is anticipated to be useful for future investigations into molecular design.

#### Interactions with mutants with defective membrane structures

Despite the fact that Gram-negative and Gram-positive bacteria have different cell wall and membrane structures, most membrane-intercalating molecules, including arylamide oligomers, polycarbonate polymers, and cationic steroid compounds, have similar antimicrobial function according to Gram-type.^11^,^46^–^48^ Mechanistic studies on cationic phenylene ethynylene oligomers and polymers additionally show that electrostatic interactions are important for the initial binding between the molecules with lipid membranes, but bacterial lipid composition can also be an important factor in determining the sensitivity of bacteria.^49^,^50^

The differences in MIC values between *E. faecalis* and *E. coli* in our study indicate that COE1 series are at least 4 times more toxic to Gram-positive bacteria as to Gram-negative bacteria (Fig. 2–4). Our observations hence indicate that: (1) COE interaction with cell membranes can vary with different cell membrane structures, and (2) COE antimicrobial mechanisms might be different from other membrane-intercalating molecules as a result of the properties of the phenylene vinylene repeat unit.

Previous studies have shown a lipid based driver for the toxicity of DSBN+, DSSN+, and 4F-DSBN+ and proposed that major differences between Gram-positive and Gram-negative microbial membranes, namely diphosphatidylglycerol (DPG) lipid content in the cell membrane as a reason for the selective toxicity of COEs towards Gram-positive bacteria.^37^ This proposed mechanism aligns well with our findings of greater toxicity of COE1 series in *E. faecalis* than on *E. coli*. We also find similar microbial sensitivities to COE1 series from *E. faecalis ΔdltA-D*, which has a more negatively charged cell surface, and the wild-type *E. faecalis* OG1X based on their MIC results (Fig. 3 and 4). This observation suggests that electrostatic interactions are playing a subordinate role in determining COE disruption on cell membranes, though they might be important for the initial binding with lipid membranes.

*E. coli* WBB06 has a more permeable outer membrane compared to both *E. coli* K-12 and W3110.^41^ The MIC results in Fig. 2–4 show that **COE1-3Py** is more toxic to both *E. coli* WBB06 (MIC = 2 μM) and *E. coli* W3110 (MIC = 4 μM) than to wild-type *E. coli* K-12 (MIC = 32 μM), while **COE1-3** has similar toxicity to K-12 and W3110 (MIC = 64 μM) and slightly greater toxicity to WBB06 (MIC = 32 μM). Among the three *E. coli* strains, WBB06 is also the most sensitive to COE1 series. COEs with short chain length and prydinium cationic groups appear more toxic to cells with increased cell membrane permeability, suggesting that the topology of pyridinium substituted molecules limits their passage through the outer membrane of *E. coli.* In light of the urgent need for new treatment options for Gram-negative infections, the increased activity against *E. coli* of **COE1-3Py** is important as is the increased activity against WBB06 as this offers significant insights into the design of future more potent compounds.^51^

#### Backbone length of the COE2 series

COE2 structures with longer backbone lengths (**COE2-Bip**, **COE2-BipF**, and **COE2-DSBT**) proved significantly less toxic than **COE2-3** (Table 3), in agreement with observations on COE1 series. In light of this emerging relationship between antimicrobial properties and molecular backbone length observed with both COE1 and COE2 series, and reasoning that two ringed structures may be expected to follow this trend, we designed a two ringed COE with alkoxy pendant groups, namely **COE2-2**. However, **COE2-2** is less toxic than its shorter three-ring homolog **COE2-3** to *E. coli* (Table 3). As discussed in previous section, UV-Vis spectroscopy and confocal microscopy indicate that **COE2-2** has a weak driving force to spontaneously intercalate into cell membranes relative to the other COE molecules in our study (Table 2, Fig. 1 and S4†).

Finally, we tried to prepare **COE1-2** ((*E*)-6,6′,6′′,6′′′-((ethene-1,2-diylbis(benzene-5,3,1-triyl))tetrakis(oxy))tetrakis(*N*,*N*,*N*-trimethylhexan-1-aminium)), which contains two aromatic rings and tertiary amine pedant groups. The resulting product was unstable, presumably due to the high electron density within the conjugated framework, and rapid decomposition into a complex mixture of products upon protonation. Practically, the three ringed configuration represents the most useful molecular configuration for achieving high toxicity in the COE1 and 2 series.

#### Backbone composition of the COE2 series

Fluorine substitutions of the **COE1-3** backbone, (previously described in the literature as 4F-DSBN+), was previously shown to result in slightly lower toxicity on *E. coli* than **COE1-3** (previously described in the literature as DSBN+).^34^ The proposed explanation is that 4F-DSBN+ has less tendency to aggregate within the bilayer than **COE1-3** (DSBN+) because the fluorine substituted distyryl benzene structure is more hydrophobic.^34^ As shown in Table 3, the fluorine substituted **COE2-3** structure, **COE2-3F**, is less toxic than **COE2-3**, with a MIC of 2048 μM for **COE2-3F** compared to an MIC of 8 μM for **COE2-3**. Antimicrobial activity was additionally attenuated by fluorine substitution in **COE2-BipF** (MIC = 2048 μM), compared with **COE2-Bip** (MIC = 512 μM), as shown in Table 3.

Confocal microscopy images in Fig. 1 confirm that **COE2-3**, **COE2-3F**, **COE2-BiP**, and **COE2-DSBT** intercalate into *E. col*i cell membranes. As quantified by UV-Vis spectroscopy, the lower toxicity of **COE2-3F** is not due to lower uptake relative to **COE2-3** (Table 3). On the contrary, more **COE2-3F** is absorbed by cells (65%) than **COE2-3** (27%). Compared to **COE2-3**, **COE2-DSBT**, which has a benzothiadiazole on its backbone, did not show improved antimicrobial properties (Table 3). This particular benzothiadiazole for phenylene substitution therefore appears to lead to less pronounced membrane disruption upon insertion into the membrane.

## Conclusion

In summary, we provide a comparison of the antimicrobial properties of twelve COE structures, including seven new molecular designs. UV-Vis absorption and PL spectroscopies were used to characterize the optical properties of COEs. These data, in combination with confocal microscopy, show that structural variations have little effect on COE uptake and intercalation into cell membranes, with the notable exception of the shortest oligomer species, namely **COE2-2**. MIC tests on both Gram-positive and Gram-negative bacteria indicate that shorter molecular backbone lengths lead to more effective antimicrobial structures with optimum practical antimicrobial activity demonstrated for three ringed compounds. Increased toxicity with pyridinium ionic groups is observed only in COEs with short backbones (three-ring). In the COE2 series, shortening the backbone length can affect the uptake of COEs by cells, thus alternating their toxic properties. Although in our studies **COE2-2** did not intercalate into cell membranes, it still exhibited comparable toxicity to the other COEs whose membrane insertion is confirmed. One intriguing possibility is that this molecular species is in fact very effective once in the membrane, but equilibrium considerations with respect to intercalated and solvated species prevent full uptake . Fluorine substitution on the COE backbones of two COE molecules resulted in significantly less antimicrobial activity. While the addition of benzothiadiazole to COE backbone showed no enhanced antimicrobial function compared to **COE2-3**. We also found that COEs with prydinium cationic groups are more toxic relative to their tetraalkyammonium counterparts to *E. coli* WBB06, which has increased cell membrane permeability than its parental type W3110, suggesting that the insertion of COEs within the inner cytoplasmic membrane is important in their antimicrobial activity. In contrast to the antimicrobial profiles reported for other membrane-intercalating molecules, such as cationic phenylene ethynylene oligomers and polymers, COEs show greater toxicity to *E. faecalis* than to *E. coli*, pointing to a different mechanism of membrane intercalation and disruption by these special phenylenevinylene backbone structures. The findings included here provide a systematic study of COEs structure for antimicrobial activity and provide insights into the molecular design of a new class of antimicrobial compounds.

## Funding sources

Funding was provided by the Institute for Collaborative Biotechnologies (ICB) under Grant No. W911F-09-D-0001 from the U.S. Army Research Office. SCELSE is funded by Singapore's National Research Foundation, Ministry of Education, Nanyang Technological University (NTU), and National University of Singapore (NUS) and is hosted by NTU in partnership with NUS. Z. D. R. is grateful for funding from the National Science Foundation Graduate Research Fellowships Program (NSF GRFP).

## Supplementary Material

Supplementary information
