# Supplementary material for: Influence of molecular structure on the antimicrobial function of phenylenevinylene conjugated oligoelectrolytes
Source: Chem Sci. 2016 Jun 1;7(9):5714–22. doi: 10.1039/c6sc00630b (PMC6021957; doi:10.1039/c6sc00630b)
Supplement: Supplementary file 1 [file SC-007-C6SC00630B-s001.pdf]

Electronic Supplementary Material (ESI) for Chemical Science.
This journal is © The Royal Society of Chemistry 2016

Supporting Information for Molecular Structure of Conjugated Oligoelectroltyes for Antimicrobial Treatment

Hengjing Yan[i], Zachary D. Rengert[i], Alexander W. Thomas[i], Carolin Rehermann[ii], Jamie Hinks[, iii], Guillermo C. Bazan[i, iv *]*

Department of Chemistry and Biochemistry, Center for Polymers and Organic Solids, University of California Santa Barbara, Santa Barbara, CA, USA; Deparment of Chemistry, Ludwig-Maximilians-Universität München; Singapore Centre on Environmental Life Sciences Engineering, Nanyang Technological University, Singapore; Department of Materials, University of California Santa Barbara, Santa Barbara, CA, USA;[v]King Abdulaziz University Jeddah, Saudi Arabia

E-mail: bazan@chem.ucsb.edu

*To whom correspondence should be addressed
[i]Department of Chemistry and Biochemistry, Center for Polymers and Organic Solids, University of California Santa Barbara, Santa Barbara, CA, USA
[ii]Deparment of Chemistry, Ludwig-Maximilians-Universität München
[iii]Singapore Centre on Environmental Life Sciences Engineering, Nanyang Technological University, Singapore
[iv]Department of Materials, University of California Santa Barbara, Santa Barbara, CA, USA

Contents

S1 Detailed Synthesis 5
S1.1 Synthesis of 3,5-bis(6-iodohexyloxy)benzaldehyde (2) 5
S1.2 Synthesis of 1,4-bis(bromometyhl)-2,3,5,6-tetrafluorobenzene (6) 6
S1.3 Synthesis of tetraethyl((perfluoro-1,4-phenylene)bis(methylene))bis (phosphonate) (7) 6
S1.4 Synthesis of 4,4`-bis(bromomethyl)-2,2`,3,3`,5,5`,6,6`-octofluoro-1,1`-biphenyl (9) ... 7
S1.5 Synthesis of tetraethyl((perfluoro-[1,1`-biphenyl]-4,4`diyl) bis(methylene))bis(phosphonate) (10) 7
S1.6 Synthesis of 5,5`-((1E,1`E)-(perfluoro-1,4-phenylene)bis(ethene-2,1-diyl))bis(1,3-bis((6-iodohexyl)oxy) benzene (COE2-3NF) 8
S1.7 Synthesis of 4,4`-Bis((E)-3,5-bis((6-iodohexyl)oxy)styryl)-2,2`,3,3`,5,5`,6,6`-octafluoro-1,1`-biphenyl (COE2-BiPNF) 9
S1.8 Synthesis of tetraethyl ([1,1'-biphenyl]-4,4'-diylbis(methylene))bis (phosphonate) (12) 10
S1.9 Synthesis of 4,4`-bis((E)-3,5-bis((6-iodohexyl)oxy)styryl) -1,1`-biphenyl (COE2-BiPN) (13) 11
S1.10 Synthesis of 1,3-bis(5-iodohexoxy)-5-vinylbenzene (14) 12
S1.11 Synthesis of (E)-1,2-bis(3,5-bis(6-iodohexoxy)phenyl)ethane (COE2-2N) (15) 13
S1.12 Synthesis of 4,7-bis((E)-3,5-bis((6-iodohexyl)oxy)styryl)benzo [c][1,2,5]thiadiazole (COE2-DSBTN) (16) 14
S1.13 Quaternization of neutral COE´s 15
S2 Organisms and Culture Conditions 18
S2.1 Summary of the organisms used in this study 18
S3 Quantification of COE uptake by *E. coli* K-12 cells 19
S3.1 Cell uptake Experiment 19
S3.2 Quantification of COE concentrations using UV-vis spectroscopy 19
S3.3 UV-vis spectroscopy of the cell uptake experiment on COEs 20
S3.4 Quantification of COE uptake percentages by *E.coli K-12* cells 23
S4 Microscopy Images of COE Treated Cells 24
References 26

List of Figures

Figure S1. Absorbance (solid curves) and fluorescence (dashed curves) scanning results of 12 COE molecules using a UV-vis plate reader (Tecan M220 Infinite Pro). E.coli-C: stained *E. coli* K-12 cells resuspended in 1×PBS buffer; E.coli-S: supernatant of 5 μM COE in 1×PBS buffer after *E. coli* K-12 cells has been added for 1.5 hours at room temperature. Fluorescence signals were collected at the maximum excitation wavelength of each COE molecule as listed in **Table-1**........20

Figure S2. Absorbance (solid curves) and fluorescence (dashed curves) scanning results of the standards of 12 COE molecules at 0, 2.5, 5, 10 μM in 1xPBS solution, using a UV-vis plate reader (Tecan M220 Infinite Pro). Fluorescence signals were collected at the maximum excitation wavelength of each COE molecule as listed in **Table-1**........21
22

Figure S3. Absorbance (solid curves) and fluorescence (dashed curves) scanning results of unstained *E. coli* K-12 cells in 1×PBS buffer for 1.5 hours at room temperature (Ecoli-C) and the supernatant from the former solution (Ecoli-S) as background references, using a UV-vis plate reader (Tecan M220 Infinite Pro). No COEs were added to those samples, labels of COE names in each panel of this figure is to indicate that the fluorescent signal of these two reference samples were collected at the same excitation wavelengths used in the corresponding panels in **Figure S1** and **Figure S2**........22

Figure S4. Confocal microscopy images of *E. coli* K-12 cells stained by COE1 series (5μM) for 1.5 hours in PBS buffer at room temperature. Scaled bar is universe for six panels. (Olympus Fluoview 1000 Spectral Confocal, Laser wavelength = 405 nm)........24

Figure S5. Microscopy images of *E. coli* K-12 cells treated by 5μM of COE2-BipF, COE2-3, and COE2-2 for 1.5 hours in PBS buffer at room temperature. Auto-fluorescent signals from *E. coli* cells were collected with the same detection settings for COE2-2 panel. Scaled bar is universe for six panels. (Olympus DSU (Spinning Disk) Confocal, Laser wavelengths 310 nm - 380 nm).........25

List of Tables

Table S1. Microorganisms and strains used in this study. 18

S1 Detailed Synthesis

The detailed synthetic procedures and characterization of final compounds and intermediates is provided below, and are shown in **scheme 1** of the main text. Unless otherwise noted, materials were purchased from suppliers (Sigma Aldrich, Acros, Strem, and TCI) and were used without further purification. ^{1}**H-** and ^{13}**C-NMR** spectra were recorded on either a Bruker DMX 500 MHz or Varian VNMRS 600 MHz spectrometer and all chemical shifts are reported in ppm values (δ) versus tetramethylsilane. Dry toluene and dry, inhibitor-free THF were taken from a solvent purification system, using packed alumina columns under Argon. Silica gel column chromatography was purchased from Dynamic Adsorbents Inc. and had particle size of 32-64 μM.

S1.1 Synthesis of 3,5-bis(6-iodohexyloxy)benzaldehyde (2)

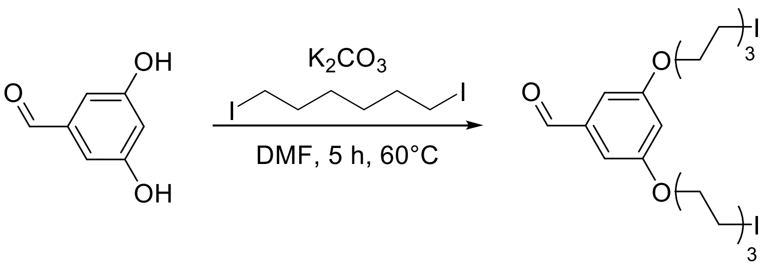

1 **2**

3,5-Dihydroxybenzaldehyde **1** (4.00 g, 28.9 mmol, 1 eq.) and 1,6-diiodohexane (36 mL, 218 mmol, 7.50 eq.) were dissolved in 15 mL DMF, heated up to 60 °C and stirred for 5 h. DMF was removed by washing with an aqueous solution of LiCl and EtOAc (3X). The combined organic layers were washed with water (2X) and brine and dried over $MgSO_4$. The solvent was removed by rotary evaporation. Silica gel chromatography (EtOAc/ hexane 1:7) was used for further purification. Aldehyde **2** was afforded as a white solid (7.52 g, 46 % yield). ^{1}**H-NMR (500 MHz, CD_2Cl_2):** δ 9.88 (s, 1H), 6.97 (m, 2H), 6.70 (m, 1H), 4.00 (t, 4H), 3.22 (t, 4H), 1.80 (m, 8H), 1.50 (m, 8H).

S1.2 Synthesis of 1,4-bis(bromometyhl)-2,3,5,6-tetrafluorobenzene (6)

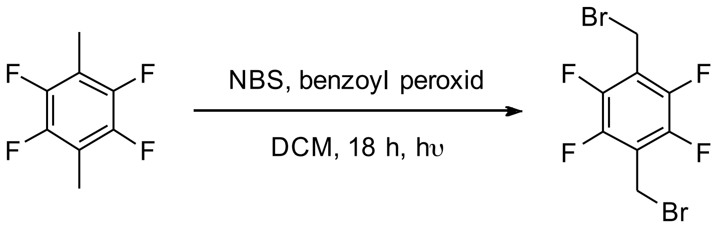

5 6

1,2,4,5-Tetrafluoro-3,6-dimethylbenzene **5** (500 mg, 2.81 mmol, 1 eq.) was dissolved in 15 mL DCM. NBS (1.60 g, 8.99 mmol, 3.2 eq.) and benzoylperoxid (75.1 mg, 0.31 mmol, 0.11 eq.) were added to the stirring solution. The solution was illuminated with two 200 W lamps for 18 h. The organic phase was washed with water (3X), dried over $MgSO_4$ and the solvent was removed by rotary evaporation. The product **6** was afforded as a colorless solid (789 mg, 83 % yield). **^{1}H-NMR (500 MHz, CD_2Cl_2):** δ 4.50 (s, 4H).

S1.3 Synthesis of tetraethyl((perfluoro-1,4-phenylene)bis(methylene))bis (phosphonate) (7)

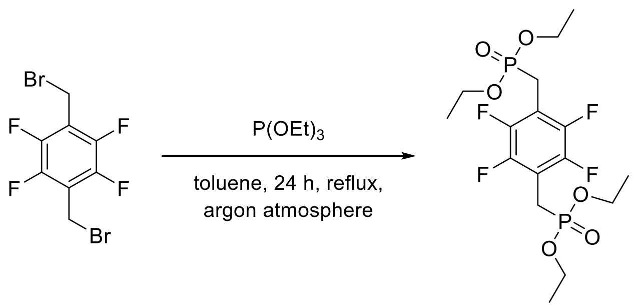

6 7

Compound **6** (500 mg, 1.48 mmol, 1 eq.) and triethylphosphite (0.56 mL, 3.28 mmol, 2,2 eq.) were dissolved in 3 mL toluene. The solution was heated up to reflux under argon atmosphere for 24 h. The solvent was removed via rotary evaporation and the crude was washed with hexane (2X) and dried overnight under vacuum. The phosphonate **7** was afforded as a colorless solid

(476 mg, 71 % yield). **^{1}H-NMR (500 MHz, CD_2Cl_2):** δ 4.11 (m, 4H), 3.25 (d, 4H), 1.29 (t, 12H).

S1.4 Synthesis of 4,4`-bis(bromomethyl)-2,2`,3,3`,5,5`,6,6`-octofluoro-1,1`-biphenyl (9)

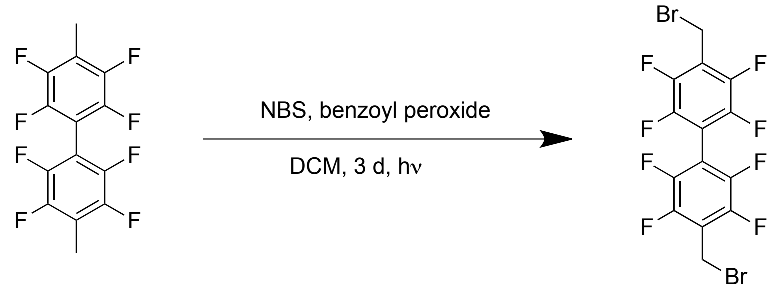

Compound **8** (500 mg, 1.53 mmol, 1 eq.) was dissolved in 15 mL DCM. NBS (820 mg, 4.16 mmol, 3 eq.) and benzoylperoxid (62.6 mg, 0.26 mmol, 0.17 eq.) were added to the stirring solution. The mixture was illuminated with two 200 W lamps for 3 d. The organic phase was washed with water (3X) and dried over $MgSO_4$. The solvent was removed via rotary evaporation. Silica gel chromatography (hexane) was used for further purification. The product **9** was afforded as a white solid (453 mg, 61 % yield). **^{1}H-NMR** (500 MHz, CD_2Cl_2): δ 4.58 (s, 4H). **FI^+-MS:** 483 (M^+), 402 $(M -Br)^+$.

S1.5 Synthesis of tetraethyl((perfluoro-[1,1`-biphenyl]-4,4`diyl) bis(methylene))bis(phosphonate) (10)

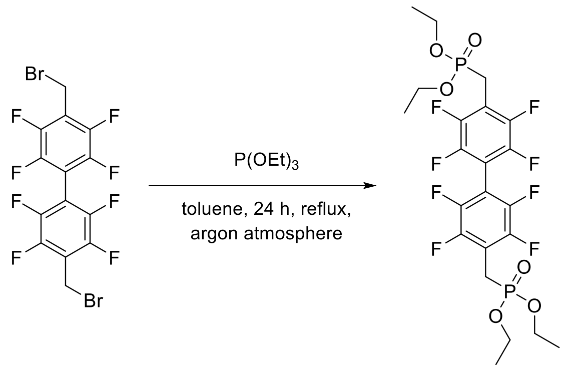

9 10

Compound **9** (300 mg, 0.62 mmol, 1 eq.) and triethylphosphite (0.25 mL, 1.37 mmol, 2.2 eq.) were dissolved in 6.2 mL toluene and heated up to reflux under argon atmosphere for 24 h. The solvent was removed via rotary evaporation and distillation with a *Kugelrohr*. For further purification the crude was washed with hexane (2X). The product **10** was afforded as a white solid (152 mg, 41 % yield). [1]**H-NMR (500 MHz, CD_2Cl_2):** δ 4.17 (m, 8H), 3.35 (d, 4H), 1.33 (t, 12H).

S1.6 Synthesis of 5,5`-((1E,1`E)-(perfluoro-1,4-phenylene)bis(ethene-2,1-diyl))bis(1,3-bis((6-iodohexyl)oxy) benzene (COE2-3NF)

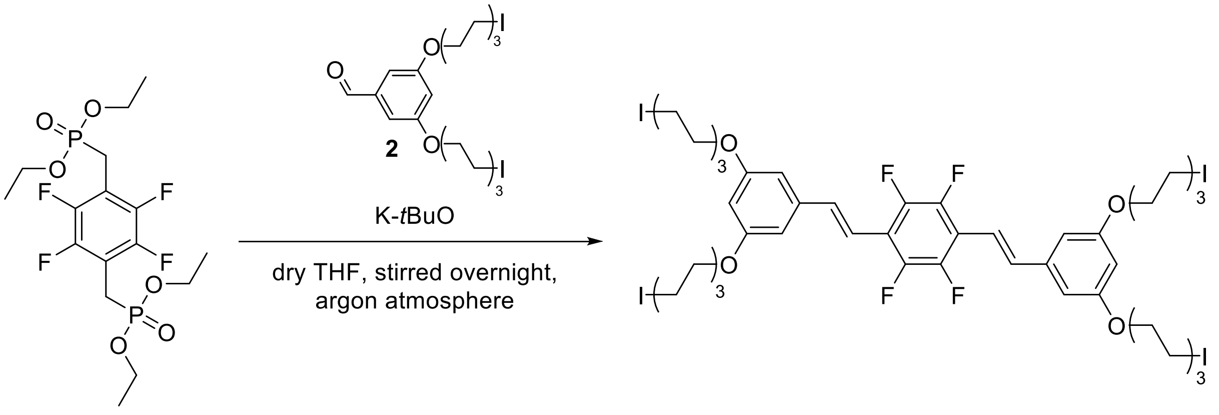

7 COE2-3NF

The bis phosphonate derivative **7** (175 mg, 0.38 mmol, 1 eq.) and Aldehyde **2** (500 mg, 0.895 mmol, 2.3 eq.) were loaded in a *Schlenk* flask, placed under argon atmosphere and dissolved in 10 mL dry THF. In a glovebox, potassium tert-butoxide (78 mg, 0.70 mmol, 1.8 eq.) was sealed inside a *Schlenk* flask, extracted from the glovebox and dissolved in 10 mL dry THF. The solution of potassium tert-butoxide was added to the mixture of **7** and **2** via a cannula. The resulting solution was stirred overnight at RT. The solvent was removed via rotary evaporation and the crude was dissolved in DCM. The organic phase was washed with water (2X) and brine and dried over $MgSO_4$. The solvent was removed and the crude was further purified by silica gel chromatography (DCM/ hexane 11:9). The product **COE2-3NF** was afforded as a light yellow solid (257 mg. 53 % yield). [1]**H-NMR** (500 MHz, CD_2Cl_2): δ 7.23 (d, 2H), 7.22 (d, 2H),

6.69 (d, 4H), 6.44 (t, 2H), 3.99 (t, 8H), 3.22 (t, 8H), 1.87 (m, 8H), 1.80 (m, 8H), 1.49 (m, 16H). ^{13}**C NMR** (600 MHz, $CDCl_3$) δ 160.45, 145.47-141.86 (m), 138.57, 136.98, 116.99, 114.87, 105.50, 101.97, 67.88, 33.40, 30.27, 29.08, 25.09, 7.03. **FD$^+$-MS:** 1258 (M^+), 1130 (M –I)$^+$, 630 (M^{2+}).

S1.7 Synthesis of 4,4`-Bis((E)-3,5-bis((6-iodohexyl)oxy)styryl)-2,2`,3,3`,5,5`,6,6`-octafluoro-1,1`-biphenyl (COE2-BiPNF)

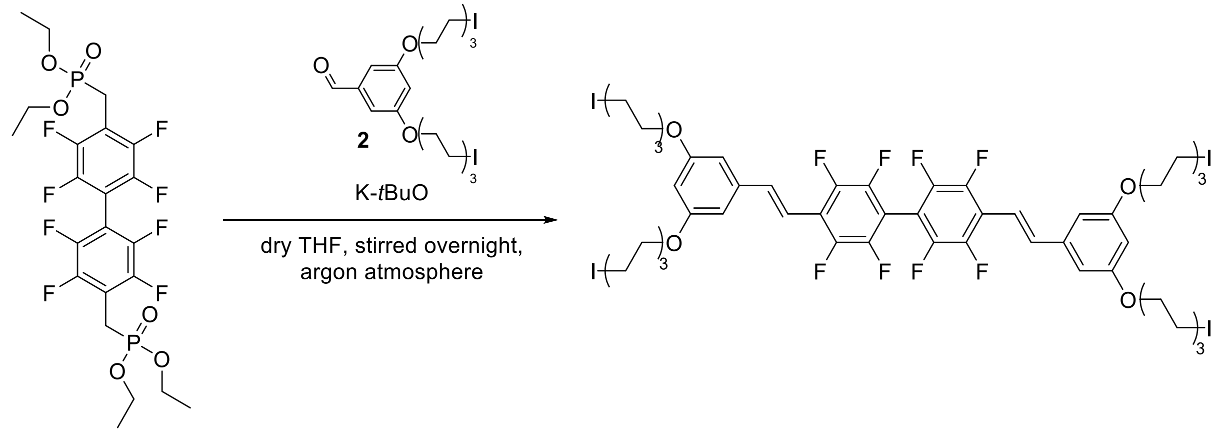

10 **COE2-biph-3NF**

Compound **10** (100 mg, 0.17 mmol, 1 eq.) and aldehyde 2 (215 mg, 0.381 mmol, 2.3 eq.) were loaded in a *Schlenk* flask, placed under argon atmosphere and dissolved in 10 mL dry THF. In a glovebox potassium tert-butoxide (78 mg, 0.70 mmol, 1.8 eq.) was sealed inside a *Schlenk* flask, extracted from the glovebox and dissolved in 10 mL dry THF. The solution of potassium tert-butoxide was added to the mixture of **4** and **2** using a cannula. The resulting solution was stirred overnight at RT. The solvent was removed via rotary evaporation and the crude was dissolved in DCM. The organic phase was washed with water (2X) and brine and dried over $MgSO_4$. The solvent was removed and the crude was further purified by silica gel chromatography (DCM/ hexane 2:1). The product **COE2-3NF** was afforded as a light yellow solid (112 mg, 47 % yield). ^{1}**H-NMR** (500 MHz, CD_2Cl_2): δ 7.29 (d, 2H), 7.28 (d, 2H), 6.69 (d, 4H), 6.45 (t, 2H), 3.99 (t, 8H), 3.21 (t, 8H), 1.87 (m, 8H), 1.81 (m, 8H), 1.51 (m, 16H). ^{13}**C-NMR** (600 MHz, $CDCl_3$) δ 156.55, 141.76 – 140.71 (m), 140.02 – 138.98 (m), 134.63 (t, J=8.5), 134.23, 114.65

(t, J=13.1), 109.77, 101.73, 100.96 (d, J=18.3), 98.32, 63.96, 29.48, 26.34, 25.77, 25.14, 21.16, 3.01. **FD$^+$-MS:** 1406 (M^+), 1278 $(M-I)^+$.

S1.8 Synthesis of tetraethyl ([1,1'-biphenyl]-4,4'-diylbis(methylene))bis (phosphonate) (12)

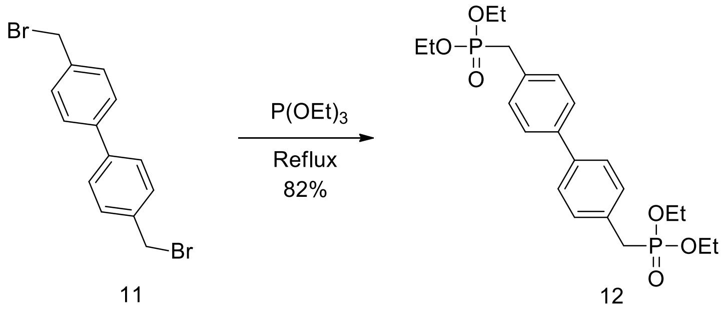

4,4'-Bis(bromomethyl)-1,1'-biphenyl **11** (1 g, 1.0eq, 2.94 mmol) was charged into a 3-neck, 25 mL round bottom flask and 1.11 mL of triethylphosphite (2.2eq, 6.47 mmol) was added, followed by 10 mL of toluene. The mixture was refluxed in toluene overnight and cooled to room temperature, at which time monitoring of the reaction by TLC showed consumption of compound **11**. After removal of solvent *in vacuo*, the resulting white solid was triturated with hexane two times to afford 1.10 g of pure **12** in 82% yield. ^{1}**H-NMR** (600 MHz, $CDCl_3$) δ = 7.48 (d, J=7.8, 2H), 7.31 (dd, J=8.1, 2.6, 2H), 3.99 (pd, J=7.2, 3.1, 4H), 3.14 (d, J=21.6, 2H), 1.21 (t, J=7.1, 6H). ^{13}**C-NMR** (600 MHz, $CDCl_3$) δ = 139.18 (d, J=3.3), 130.64 (d, J=9.2), 130.11 (d, J=6.6), 127.00 (d, J=3.0), 62.10 (d, J=6.7), 33.37 (d, J=138.1), 16.34 (d, J=5.9). **FD$^+$-MS:** 454 (M^+). **HR-MS:** $(M+Na)^+$ (Theoretical: 477.1572, Observed: 477.1558).

S1.9 Synthesis of 4,4`-bis((E)-3,5-bis((6-iodohexyl)oxy)styryl) - 1,1`-biphenyl (COE2-BiPN) (13)

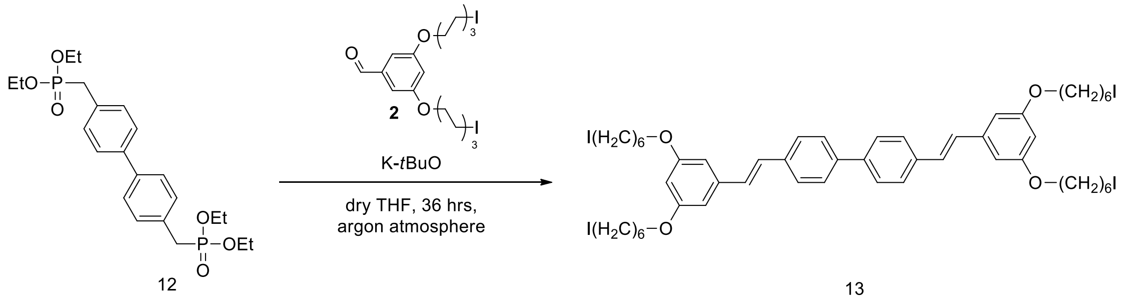

Bisphosphonate **12** (200 mg, 1.0 eq, 0.44 mmol) was combined with 565 mg of aldehyde **2** (2.3 eq, 1.01 mmol) in a 100 mL Schlenk flask and placed under an Argon atmosphere. Then 50 mL of dry THF was added to give a clear homogeneous solution. Then, 109 mg of Potassium *tert*-butoxide (2.2 eq, 0.97 mmol) dissolved in a minimal amount of dry THF under an Argon atmosphere was added dropwise *via* cannula at room temperature, the clear solution turned a light yellow color and was allowed to stir at room temperature for 36 hours, at which time TLC monitoring showed consumption of both starting materials. The resulting solution was quenched with water and the solvent was removed *in vacuo*. The resulting oil yellow oil was dissolved in ether and washed with water. The aqueous layer was back extracted with ether twice and the combine ether fractions were washed with water, brine, dried over Sodium Sulfate and concentrated *in vacuo*. The resulting yellow oil was purified with silica gel chromatography using 1:1 (v/v) dichloromethane in hexane to give 468 mg of a yellow solid (84% yield). **^{1}H-NMR** (600 MHz, $CDCl_3$) δ = 7.62 – 7.56 (dd, J=31.1,8.3, 4H), 7.08 (q, J=16.2, 4H), 6.67 (d, J=2.1, 4H), 6.39 (t, J=2.2, 2H), 3.98 (t, J=6.4, 8H), 3.20 (t, J=7.0, 8H), 1.89 – 1.78 (m, 16H), 1.55 – 1.46 (m, 16H). **^{13}C-NMR** (600 MHz, $CDCl_3$) δ 160.41, 139.70, 139.22, 136.29, 128.80,

128.52, 127.04, 127.03, 105.17, 100.98, 67.83, 33.42, 30.27, 29.11, 25.12, 7.07. **FD$^+$-MS:** 1262 (M^+), 1262 ($(M\text{-}I\text{-}CH_2)^+$), 631 ($M^{2+}$).

S1.10 Synthesis of 1,3-bis(5-iodohexoxy)-5-vinylbenzene (14)

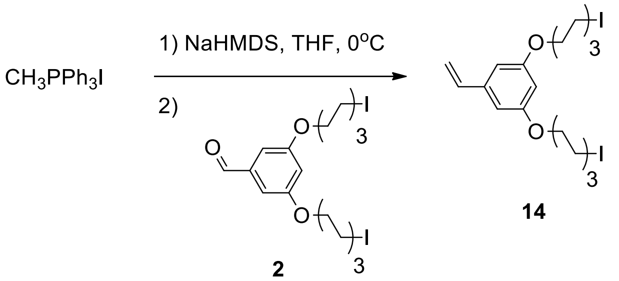

To a slurry of 837 mg of methyltriphenylphosphonium iodide (1.2 eq, 2.15 mmol) in 50 mL of dry THF at 0°C in a 3-neck 100 mL round bottom flask, was added dropwise 0.99 mL of sodium bis(trimethylsilyl)amide (1.1 eq, 2.0 M solution, 1.97 mmol). The resulting solution turned yellow and was partially homogenized over 15 minutes. Then, a 1 g solution of aldehyde **2** (1.0 eq, 1.79 mmol) in 20 mL of dry THF was added dropwise *via* cannula at 0°C and allowed to warm to room temperature. After stirring for 2.5 hrs, aldehyde **2** was consumed as monitored by TLC. The resulting solution was quenched with 0.2 mL of water, stirred for 5 minutes, and then diluted with ethyl acetate. The resulting organic phase was washed with brine, back extracted, washed again with brine, and dried over sodium sulfate. After removal of solvent *in vacuo*, the resulting pale yellow oil was purified with silica gel chromatography using 5% ethyl acetate (v/v) in hexane to give 812 mg of an off white solid (82% yield). **^{1}H-NMR** (600 MHz, $CDCl_3$) δ = 6.61 (dd, J=17.5, 10.8, 1H), 6.53 (d, J=2.2, 2H), 6.35 (t, J=2.2, 1H), 5.70 (dd, J=17.6, 0.9, 1H), 5.22 (dd, J=10.8, 0.8, 1H), 3.94 (t, J=6.4, 4H), 3.19 (t, J=7.0, 4H), 1.85 (p, J=7.0, 4H), 1.80 – 1.74 (p, J=7.0,4H), 1.47 (m, 8H). **^{13}C-NMR** (500 MHz, $CDCl_3$) δ 160.28, 139.49, 136.87,

114.16, 104.85, 100.96, 67.75, 33.38, 30.22, 29.05, 25.07, 6.95. **FD$^+$-MS:** 556 (M^+). **HR-MS:** $(M+Na)^+$ (Theoretical: 579.0233, Observed: 579.0217).

S1.11 Synthesis of (E)-1,2-bis(3,5-bis(6-iodohexoxy)phenyl)ethane (COE2-2N) (15)

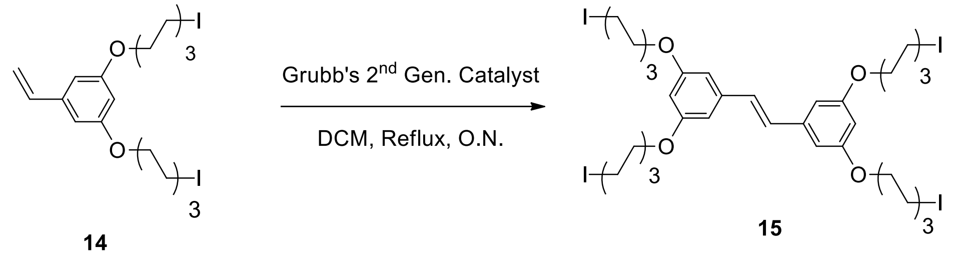

To a 2-neck 10 mL round bottom flask equipped with a reflux condenser, was added 400 mg of styrene **14** (1.0 eq, 0.72 mmol) and the apparatus was placed under an argon atmosphere *via* vacuum/argon cycling using a Schlenk line. Dry dichloromethane was added via syringe, followed by 12 mg of Grubb's 2nd generation catalyst (2 mole %, 0.014 mmol) dissolved in a minimal amount of dry toluene under argon. The resulting red solution was allowed to reflux under argon overnight. It is noteworthy to mention that the starting material and product of this reaction have the same R_f value on TLC using 10% (v/v) ethyl acetate in hexane! The resulting red solution was diluted with DCM and filtered through a silica plug. Recrystallization in a mixture of ethanol and ethyl acetate, followed by another recrystallization from acetone afforded 166 mg of stilbene **15** (43% yield). **^{1}H-NMR** (500 MHz, $CDCl_3$) δ = 7.01 (s, 2H), 6.66 (d, J=2.2, 4H), 6.40 (t, J=2.2, 2H), 4.01 (t, J=6.4, 8H), 3.24 (t, J=7.0, 8H), 1.90 (p, J=7.0, 8H), 1.83 (p, J=6.6, 8H), 1.52 (m, 16H). **^{13}C NMR** (600 MHz, $CDCl_3$) δ 160.36, 139.07, 129.09, 105.17,

100.99, 67.79, 33.38, 30.22, 29.06, 25.08, 6.96. **FD$^+$-MS:** 1084 (M$^+$). **HR-MS:** (M +Na)$^+$ (Theoretical: 1107.0255, Observed: 1107.0261).

S1.12 Synthesis of 4,7-bis((E)-3,5-bis((6-iodohexyl)oxy)styryl)benzo [c][1,2,5]thiadiazole (COE2-DSBTN) (16)

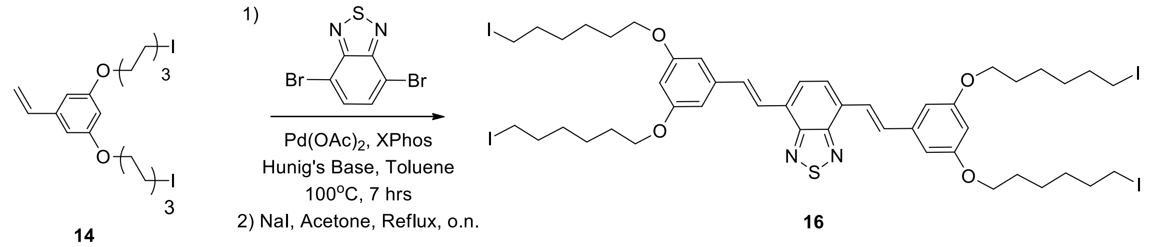

In an nitrogen filled glovebox, a 2 mL microwave tube was charged with 208 mg of styrene **14** (2.2 eq, 0.37 mmol), 50 mg of 4,7-dibromobenzo[c][1,2,5]thiadiazole (1.0 eq, 0.17 mmol), 4 mg palladium(II) acetate (10 mole %, 0.017 mmol), 16 mg XPhos (20 mole %, 0.034 mmol), 0.12 mL of Hunig's base (4 eq, 0.68 mmol), and 1 mL of toluene. The microwave tube was capped and the solution stirred for 5 min. Then, the solution was brought out of the glovebox and placed in a pre-heated oil bath at 100°C for 7 hrs, at which time the starting materials had been consumed as monitored by TLC. After cooling to room temperature, the solution was filtered through a plug of silica using DCM and the solvent removed *in* vacuo. The resulting orange solid was subjected to silica gel chromatography using 50% (v/v) chloroform in hexane to give an orange solid. The solid was dissolved in acetone and refluxed in the presence of excess sodium iodide overnight. The solution was diluted with excess DCM and the resulting orange heterogeneous solution filtered through a silica plug. The solid was triturated with ethanol twice

to give 76 mg of **16** (58% yield over two steps). ^{1}**H-NMR** (500 MHz, $CDCl_3$) δ = 7.92 (d, J=16.3, 2H), 7.68 (s, 2H), 7.61 (d, J=16.3, 2H), 6.80 (d, J=2.3, 4H), 6.44 (t, J=2.2, 2H), 4.03 (t, J=6.3, 8H), 3.24 (t, J=7.0, 8H), 1.94 – 1.81 (m, 16H), 1.54 (m, 16H). ^{13}**C NMR** (600 MHz, $CDCl_3$) δ 160.40, 153.84, 139.31, 133.29, 129.18, 127.07, 124.76, 105.46, 101.55, 67.85, 33.41, 30.27, 29.10, 25.11, 7.02. **FD$^+$-MS:** 1224 (M^+), 1131 $(M\text{-}I)^+$.

S1.13 Quaternization of neutral COE´s

COE2-3C

COE2-3N (200 mg, 0.17 mmol, 1 eq.) was dissolved in dry THF and a large excess of 3.2 molar trimethylamine solution in methanol (~6 mL) was added. The resulting solution was stirred for 24 h. The solvent was removed by rotary evaporation. The crude was dissolved in methanol and a large excess of the 3.2 molar trimethylamine solution was added before stirring for 2 d. The solvent was removed via rotary evaporation. For better handling the product was dissolved in water and freeze dried. The product **COE2-3C** was afforded as a light yellow solid (232 mg, 96 % yield).

COE2-3CF

COE2-3NF (200 mg, 0.15 mmol, 1 eq.) was used for the same quaternization reaction as described above for **COE2-3N**. The product **COE2-3CF** was afforded as a light yellow solid (203 mg, 90 % yield). ^{1}**H-NMR (500 MHz, DMSO):** δ 7.22 (d, 2H), 7.18 (d, 2H), 6.84 (d, 4H), 6.49 (t, 2H), 4.03 (t, 8H), 3.34 (m, 8H), 3.08 (s, 36H), 1.74 (m, 16H), 1.50 (m, 8H), 1.36 (m, 8H). ^{13}**C-NMR** (600 MHz, CD_3OD:DMSO(d_6)) δ 161.34, 146.00, 144.42, 139.18, 138.08, 114.59, 106.21, 102.96, 68.54, 66.82, 53.26, 29.54, 26.49, 26.13, 23.27. **ESI-MS:** 1367 $(M –I)^+$, 620 $(M\ \text{-}2I)^{2+}$,371 $(M\ \text{-}3I)^{3+}$, 246 $(M –4I)^{4+}$. **HR-MS:** $(M\ \text{-}2I)^{2+}$ (Theoretical: 620.2645, Observed: 620.2653).

COE2-BiPF

COE2-BiPNF (200 mg, 0.14 mmol, 1 eq.) was used for the same quaternization reaction as described above for **COE2-3N**. The product **COE2-BiPF** was afforded as a light yellow solid (221 mg, 94 % yield). ^{1}**H-NMR (500 MHz, DMSO):** δ 7.36 (d, 2H), 7.35 (d, 2H), 6.88 (d, 4H), 6.51 (t, 2H), 4.03 (t, 8H), 3.31 (m, 8H), 3.06 (s, 36H), 1.74 (m, 16H), 1.49 (m, 8H), 1.37 (m, 8H). ^{13}**C-NMR** (600 MHz, CD_3OD:DMSO(d_6)) δ 159.84, 145.22 – 143.46 (m), 143.46 – 141.84 (m), 137.97, 137.27, 117.98, 112.40, 104.77, 103.69, 101.69, 67.12, 65.47, 51.90, 28.05, 24.97, 24.60, 21.86. **ESI-MS:** 694 $(M\text{ -}2I)^{2+}$, 420 $(M\text{ -}3I)^{3+}$, 283 $(M\text{ -}4I)^{4+}$. **HR-MS:** $(M\text{ -}2I)^{2+}$ (Theoretical: 694.2613, Observed: 694.2615).

COE2-BiP

COE2-BiPN (206 mg, 0.16 mmol, 1 eq.) was used for the same quaternization reaction as described above for **COE2-3N**. The product **COE2-BiP** was afforded as a light yellow solid (232 mg, 95 % yield). ^{1}**H-NMR** (600 MHz, CD_3OD:DMSO(d_6)) δ = 7.76 (s, 8H), 7.35 (d, J=16.3, 2H), 7.28 (d, J=16.3, 2H), 6.86 (d, J=2.2, 4H), 6.48 (t, J=2.1, 2H), 4.09 (t, J=6.4, 8H), 3.46 – 3.39 (m, 8H), 3.19 (s, 36H), 1.84 (ddt, J=11.8, 8.7, 5.6, 16H), 1.66 – 1.58 (m, 8H), 1.48 (p, J=7.6, 8H). ^{13}**C-NMR** (600 MHz, CD_3OD:DMSO(d_6)) δ 160.62, 139.52, 139.32, 136.64, 128.87, 128.52, 127.37, 126.84, 109.99, 105.21, 67.80, 66.25, 52.56, 28.89, 25.80, 25.49, 22.61. **ESI-MS:** 622 $(M\text{-}2I)^{2+}$. **HR-MS:** $(M\text{ -}2I)^{2+}$ (Theoretical: 622.2990, Observed: 622.2977).

COE2-2

COE2-2N (50 mg, 0.046 mmol, 1 eq.) was used for the same quaternization reaction as described above for **COE2-3N**. The product **COE2-2** was afforded as a white solid (56 mg, 92 % yield). ^{1}**H-NMR** (600 MHz, CD3OD:DMSO(d_6)) δ = 7.17 (s, 2H), 6.79 (d, J=2.1, 4H),

6.40 (q, J=4.1, 3.2, 2H), 4.04 (t, J=6.4, 8H), 3.42 – 3.37 (m, 8H), 3.15 (s, 36H), 1.83 (pd, J=7.3, 6.7, 3.8, 16H), 1.61 (p, J=7.5, 8H), 1.47 (p, J=7.7, 8H). **^{13}C-NMR** (600 MHz, CD_3OD:DMSO(d_6)) δ 160.50, 139.33, 128.90, 104.98, 67.62, 66.36, 52.37, 28.72, 25.65, 25.35, 22.54. **ESI-MS:** 533 $(M-2I)^{2+}$, 313 $(M-3I)^{3+}$, 203 $(M-4I)^{4+}$. **HR-MS:** $(M -2I)^{2+}$ (Theoretical: 533.2599, Observed: 533.2589).

COE2-DSBT

COE2-DSBTN (64 mg, 0.051 mmol, 1 eq.) was used for the same quaternization reaction as described above for **COE2-3N**. The product **COE2-DSBT**was afforded as a light yellow solid (71 mg, 93 % yield). **^{1}H-NMR** (600 MHz, DMSO(d_6)) δ = 7.18 (d, J=16.1, 2H), 7.02 (d, J=15.2, 2H), 6.83 (d, J=16.2, 2H), 5.99 (s, 4H), 5.63 (s, 2H), 3.25 (d, J=7.7, 8H), 2.64 – 2.57 (m, 8H), 2.35 (s, 36), 1.03 (dd, J=13.7, 7.1, 16H), 0.80 (q, J=8.0, 7.6, 8H), 0.67 (dd, J=14.2, 7.5, 8H). **^{13}C-NMR** (600 MHz, CD_3OD:DMSO(d_6)) δ 159.76, 152.84, 138.69, 132.45, 128.24, 126.88, 123.82, 104.33, 100.61, 66.82, 65.55, 51.59, 27.97, 24.90, 24.58, 21.75. **ESI-MS**: 243.19 $(M-4I)^{4+}$, 366.56 $(M-3I)^{3+}$, 613.29 $(M-2I)^{2+}$. **HR-MS:** $(M -2I)^{2+}$ (Theoretical: 613.2646, Observed: 613.2639).

COE1-3Py

COE1-3N (48 mg, 0.036 mmol, 1 eq.) was used for the same quaternization reaction as described above for **COE2-3N**. The product **COE1-3Py** was afforded as an orange solid (53 mg, 90 % yield). **^{1}H NMR** (600 MHz, DMSO(d_6)) δ = 8.28 (d, *J*=6.0, 8H), 7.83 (t, *J*=7.8, 4H), 7.36 (t, *J*=7.1, 8H), 6.70 (s, 4H), 6.62 (d, *J*=8.3, 4H), 6.30 (d, *J*=16.2, 2H), 6.14 (d, *J*=16.2, 2H), 5.89 (d, *J*=8.3, 4H), 3.88 (t, *J*=7.6, 8H), 2.56 (t, *J*=7.7, 8H), 1.25 (p, *J*=7.1, 8H), 0.82 (p,

J=7.4, 8H), 0.71 – 0.55 (m, 16H). **ESI-MS:** 607 $(M\text{-}2I)^{2+}$, 362 $(M\text{-}3I)^{3+}$, 240 $(M\text{-}4I)^{4+}$. **HR-MS:** $(M\text{ -}2I)^{2+}$ (Theoretical: 607.2418, Observed: 607.2416).

COE1-5Py

COE1-5N (65 mg, 0.048 mmol, 1 eq.) was used for the same quaternization reaction as described above for **COE2-3N**. The product **COE1-5Py** was afforded as an orange solid (72 mg, 90 % yield). **^{1}H NMR** (600 MHz, DMSO(d_6)) δ = 9.03 (d, *J*=5.9, 8H), 8.56 (t, *J*=7.8, 4H), 8.11 (t, *J*=7.0, 8H), 7.59 – 7.51 (m, 8H), 7.48 (d, *J*=7.6, 4H), 7.35 (d, *J*=8.4, 4H), 7.21 (d, *J*=3.9, 4H), 7.08 (d, *J*=16.1, 2H), 6.88 (d, *J*=16.1, 2H), 6.58 (d, *J*=8.2, 4H), 4.56 (t, *J*=7.5, 8H), 3.26 – 3.21 (m, 8H), 1.90 (p, *J*=7.0, 6.6, 8H), 1.48 (s, 8H), 1.30 (d, *J*=8.2, 16H). **ESI-MS:** 709 $(M\text{-}2I)^{2+}$, 430 $(M\text{-}3I)^{3+}$, 291 $(M\text{-}4I)^{4+}$. **HR-MS:** $(M\text{ -}2I)^{2+}$ (Theoretical: 709.2877, Observed: 709.2888).

S2 Organisms and Culture Conditions

S2.1 Summary of the organisms used in this study

Table S1. Microorganisms and strains used in this study.

Strain	Growth conditions	Reference or source
E. coli K-12	37°C	ATTC 10798
E. coli W3110	37°C	*E. coli* Genetic Stock Center,<br>Yale University
E. coli WBB06	37°C, 12 μg/ml tetracycline	Reference[1]
E. faecalis OG1X	37°C	Reference[2]
E. faecalis OG1X *ΔdltA-D*	37°C	Reference[3]

S3 Quantification of COE uptake by *E. coli* K-12 cells

S3.1 Cell uptake Experiment

E. coli K-12 cell culture (OD=1.0) grown overnight from single colonies in LB medium (244620; Difco, BD, USA) were collected after centrifuging for 5 minute at 8000 × *g*. Cells were washed twice with phosphate buffered saline (PBS) solution (pH=7.2) and then stained withy 5 μM COE in 1×PBS buffer for 1.5 hours at room temperature. During staining, centrifuge tubes were covered with foil to avoid possible COE interaction with light. Stained cells were separated by centrifugation for 5 minutes at 8000 × *g* and then suspended in 1×PBS buffer (OD=1.0) for UV-vis spectroscopy and OD=2.0 for confocal microscopy. The supernatant was collected for UV-vis spectroscopy to quantify COEs that was left in solution after cell uptake.

S3.2 Quantification of COE concentrations using UV-vis spectroscopy

An absorbance scan was carried out using a plate reader (Tecan M220 Infinite Pro) to obtain absorption curves for sample blanks (unstained *E. coli*), standard solution of COE, treatment supernatants samples, and stained *E. coli* resuspended in PBS. The COE concentration of each sample was calculated according to the linear relationships between the blank values and those obtained with COE standards of each COE at 0, 2.5, 5, 10 μM in PBS buffer.

S3.3 UV-vis spectroscopy of the cell uptake experiment on COEs

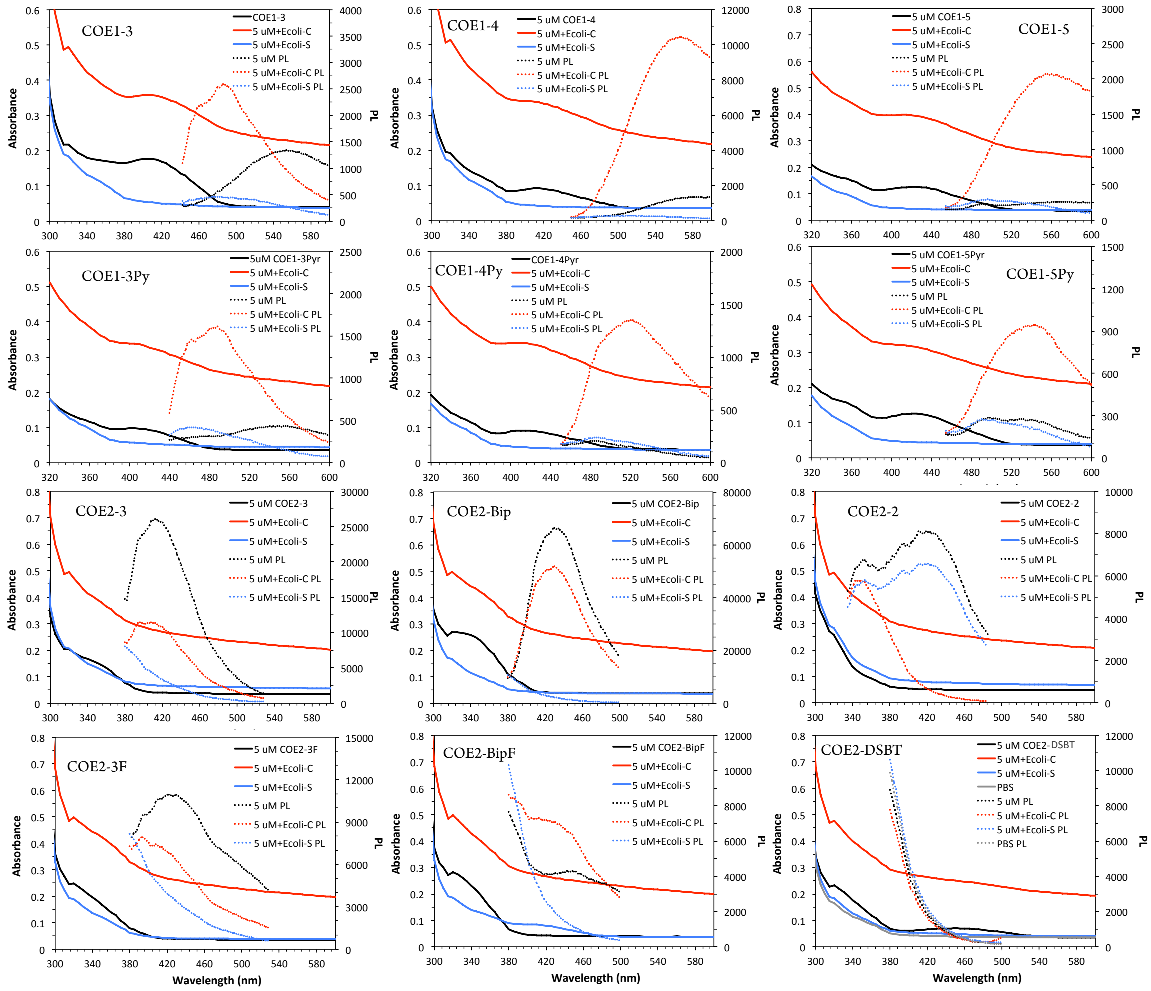

Figure S1. Absorbance (solid curves) and fluorescence (dashed curves) scanning results of 12 COE molecules using a UV-vis plate reader (Tecan M220 Infinite Pro). E.coli-C: stained *E. coli* K-12 cells resuspended in 1×PBS buffer; E.coli-S: supernatant of 5 μM COE in 1×PBS buffer after *E. coli* K-12 cells has been added for 1.5 hours at room temperature. Fluorescence signals were collected at the maximum excitation wavelength of each COE molecule as listed in **Table-1**.

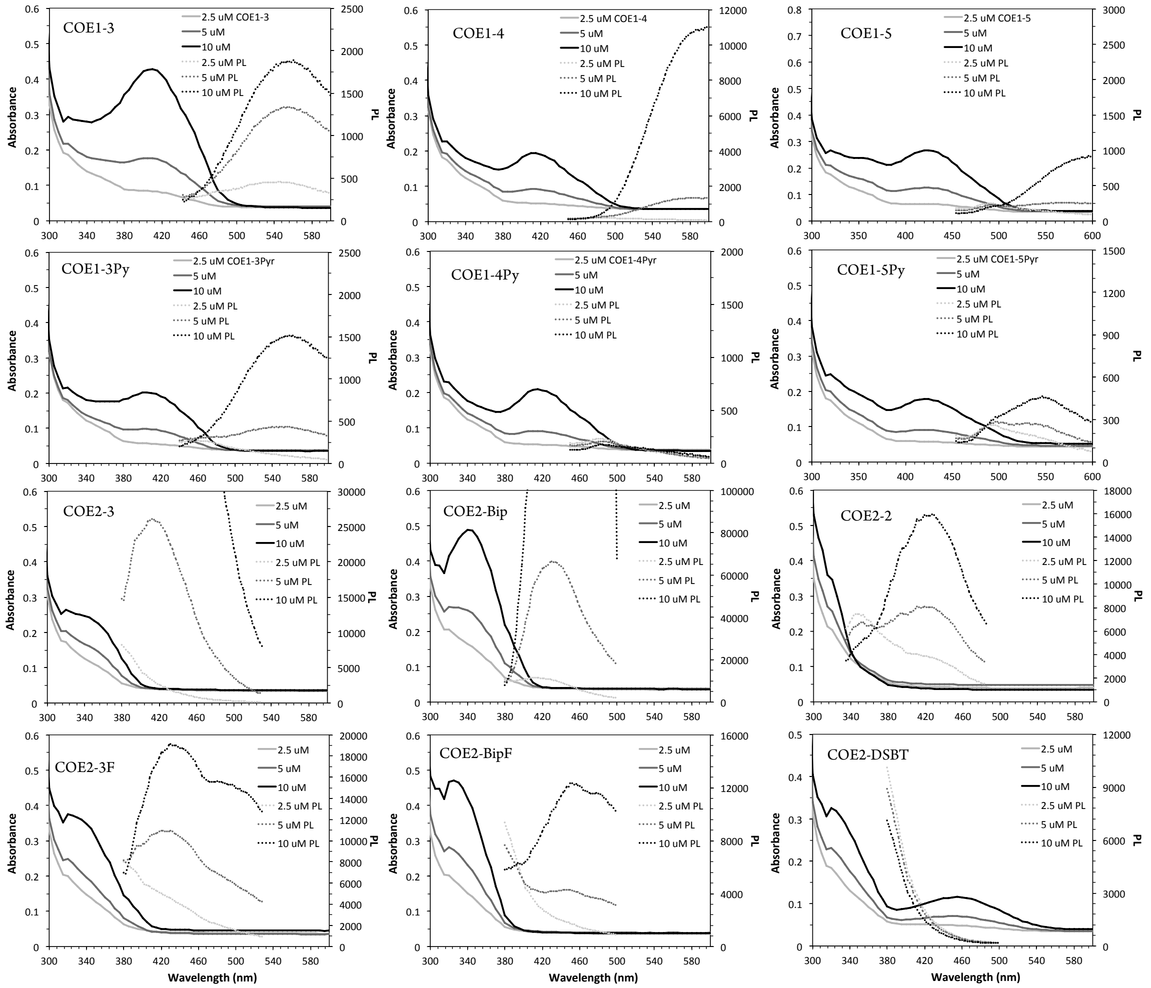

Figure S2. Absorbance (solid curves) and fluorescence (dashed curves) scanning results of the standards of 12 COE molecules at 0, 2.5, 5, 10 μM in 1xPBS solution, using a UV-vis plate reader (Tecan M220 Infinite Pro). Fluorescence signals were collected at the maximum excitation wavelength of each COE molecule as listed in **Table-1**.

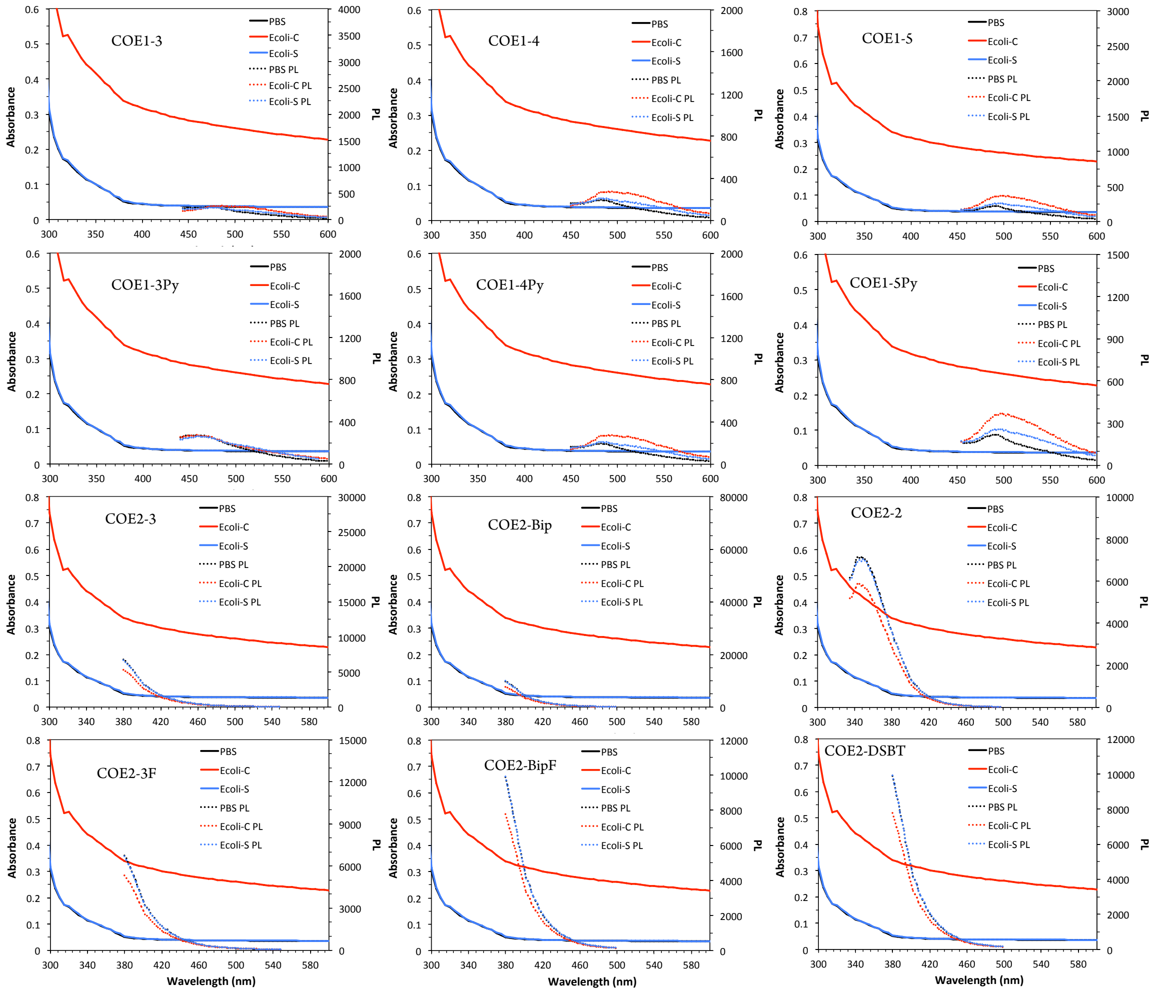

Figure S3. Absorbance (solid curves) and fluorescence (dashed curves) scanning results of unstained *E. coli* K-12 cells in 1×PBS buffer for 1.5 hours at room temperature (Ecoli-C) and the supernatant from the former solution (Ecoli-S) as background references, using a UV-vis plate reader (Tecan M220 Infinite Pro). No COEs were added to those samples, labels of COE names in each panel of this figure is to indicate that the fluorescent signal of these two reference samples were collected at the same excitation wavelengths used in the corresponding panels in **Figure S1** and **Figure S2**.

S3.4 Quantification of COE uptake percentages by *E.coli K-12* cells

Results from Figure S3 suggested that the absorbance signal from the supernatant of untreated E. coli culture (Ecoli-S) is almost identical with the absorbance signal from sterile PBS, therefore, the percentage of COEs absorbed by *E. coli* K-12 cells, x%, was calculated relative proportion of the total absorbance value of a 5 μM COE ($I_{5\mu M}$) solution of the corresponding COE and that of the supernatant (E.coli-S) samples after treatment (**Figure S1**) at the maximum absorbance wavelength of each COE molecule, using the following equation:

$$\text{x\%} = [(I_{5\mu M} - I_{5\mu M+Ecoli_S})\times 100/I_{5\mu M}]\%.$$

S4 Microscopy Images of COE Treated Cells

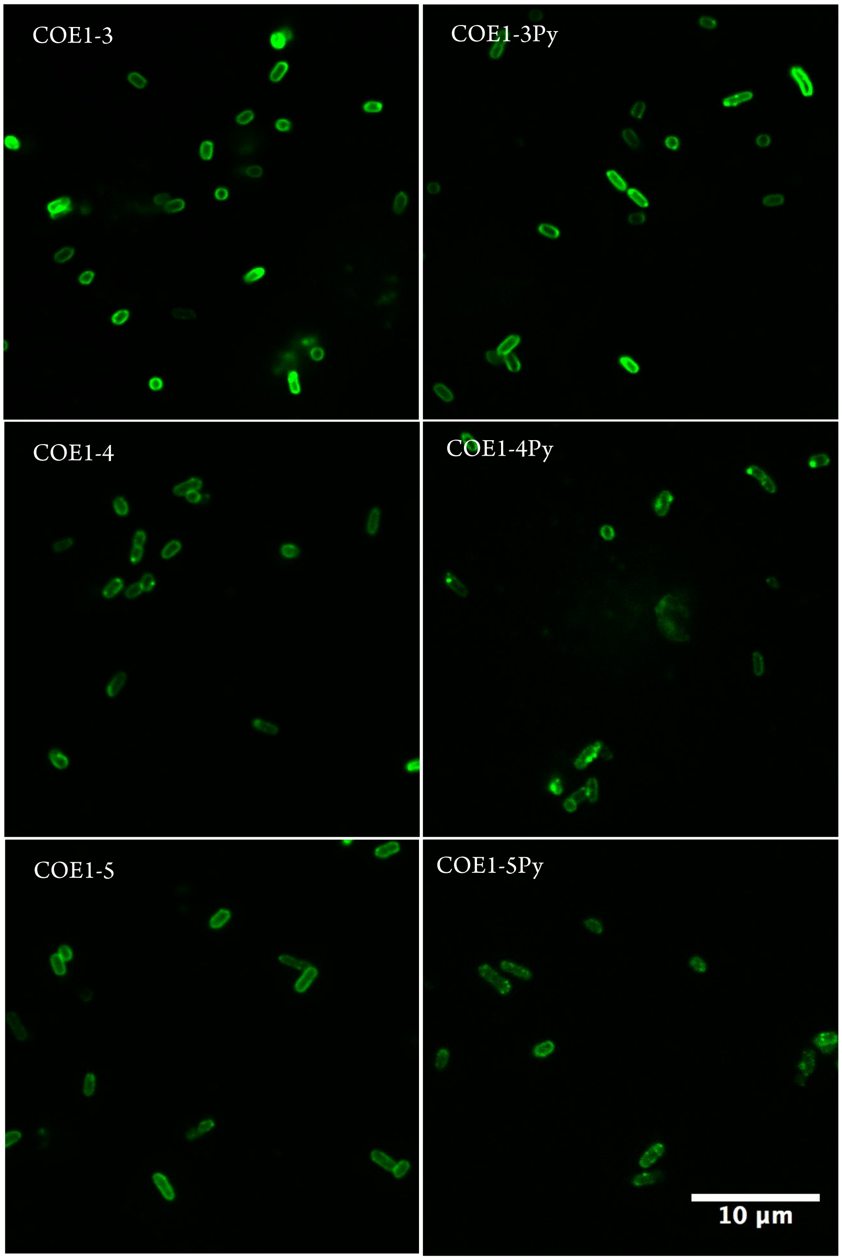

Figure S4. Confocal microscopy images of *E. coli* K-12 cells stained by COE1 series (5μM) for 1.5 hours in PBS buffer at room temperature. Scaled bar is universe for six panels. (Olympus Fluoview 1000 Spectral Confocal, Laser wavelength = 405 nm)

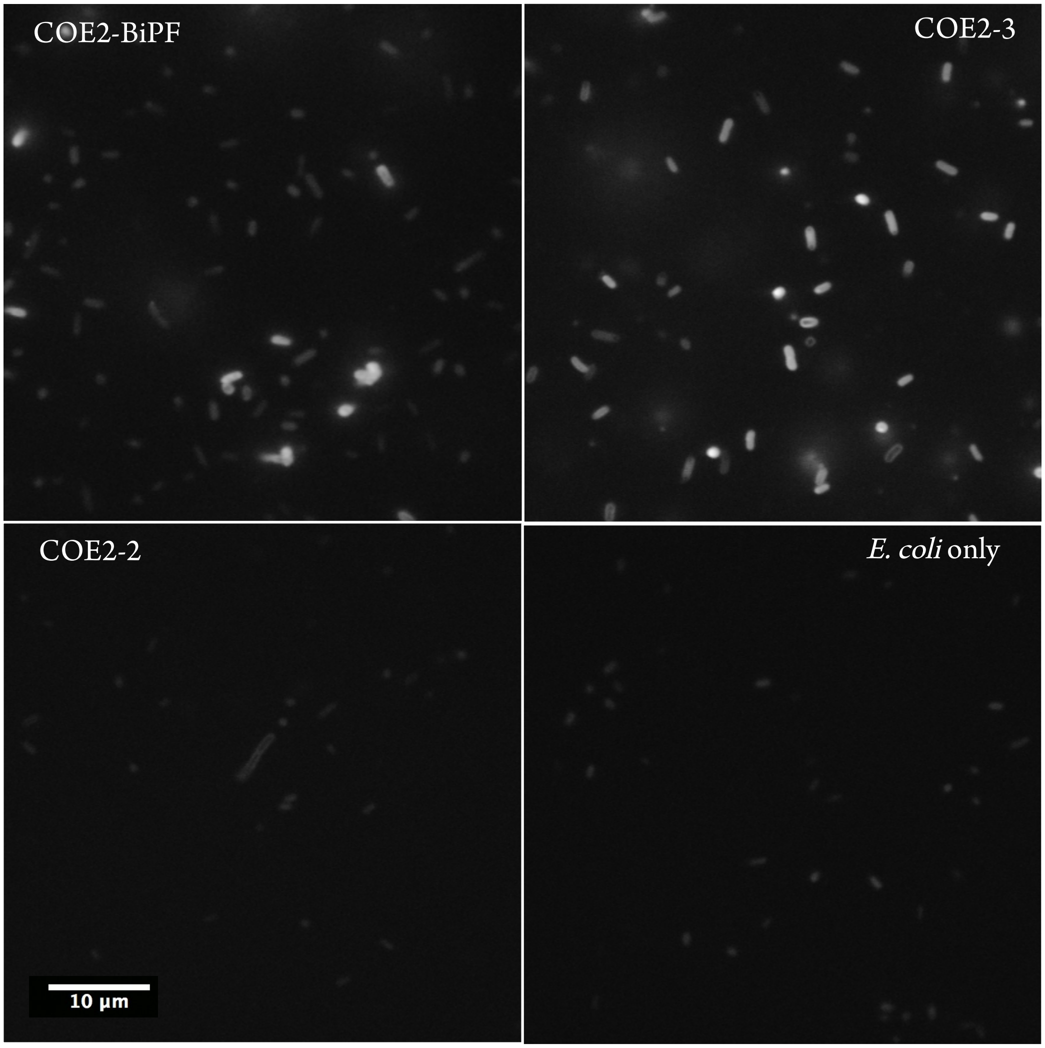

Figure S5. Microscopy images of *E. coli* K-12 cells treated by 5μM of COE2-BipF, COE2-3, and COE2-2 for 1.5 hours in PBS buffer at room temperature. Auto-fluorescent signals from *E. coli* cells were collected with the same detection settings for COE2-2 panel. Scaled bar is universe for six panels. (Olympus DSU (Spinning Disk) Confocal, Laser wavelengths 310 nm - 380 nm).

References

(1) Brabetz, W.; Muller-Loennies, S.; Holst, O.; Brade, H. *European Journal of Biochemistry* **1997**, *247*, 716.
(2) Ike, Y.; Clewell, D. B.; Segarra, R. A.; Gilmore, M. S. *Journal of Bacteriology* **1990**, *172*, 155.
(3) Hinks, J.; Han, P. W.; Chu, J. J. H.; Loo, J. S. C.; Bazan, G. C.; Hancock, L. E.; Wuertz, S. *Applied and Environmental Microbiology* **2015**.
